# Novel XAD-LC/MS-FBMN-IMS Strategy for Screening *Holo-*Hydroxamate Siderophores: Siderome Analysis of the Pathogenic Bacterium *Tenacibaculum maritimum*

**DOI:** 10.1021/acs.analchem.5c01687

**Published:** 2025-06-16

**Authors:** Lucía Ageitos, Larissa Buedenbender, M. Pilar Escribano, Abel Mateo Forero, Beatriz Santos, Miguel Balado, Manuel L. Lemos, Beatriz Magariños, Jaime Rodríguez, Carlos Jiménez

**Affiliations:** † Departmento de Química, Facultad de Ciencias and CICA - Centro Interdisciplinar de Química e Bioloxía, 16737 Universidade da Coruña, 15071 A Coruña, Spain; ‡ Department of Microbiology and Parasitology, Aquatic One Health Research Center (iARCUS), 16780 Universidade de Santiago de Compostela, 15705 Santiago de Compostela, Spain

## Abstract

Hydroxamate siderophores are key virulence factors in multiple pathogens, and their structures present interesting scaffolds for potential biotechnological applications such as the design of novel antimicrobials. However, their variable and scarce production, their low stability, and occurrence as complex mixtures in natural samples complicate their analysis. Herein, we present a new strategy, named XAD-LC/MS-FBMN-IMS, which enables the analysis of the *holo*-hydroxamate siderophore composition from microbial cultures by integrating traditional XAD resin extraction, modern Feature-Based Molecular Networking (FBMN) tools, and a novel direct infusion ion mobility mass spectrometry validation approach. Using ferrioxamine as a model, XAD resins demonstrated high efficiency in extracting hydroxamate siderophore metal complexes when compared to other sorbents. This strategy was applied for detecting the siderophores produced by *Tenacibaculum maritimum*, a relevant pathogenic bacterium in fish aquaculture. This analysis unveiled three families of hydroxamate siderophores, including three known desferrioxamine derivatives along with 17 new putative acyl-desferrioxamine-like structures. Overall, the XAD-LC/MS-FBMN-IMS strategy significantly enhances siderophore and metallophore investigation in microbial cultures, improving our understanding of their production and roles in bacteria and fungi, ultimately facilitating their biotechnological applications.

## Graphical abstract

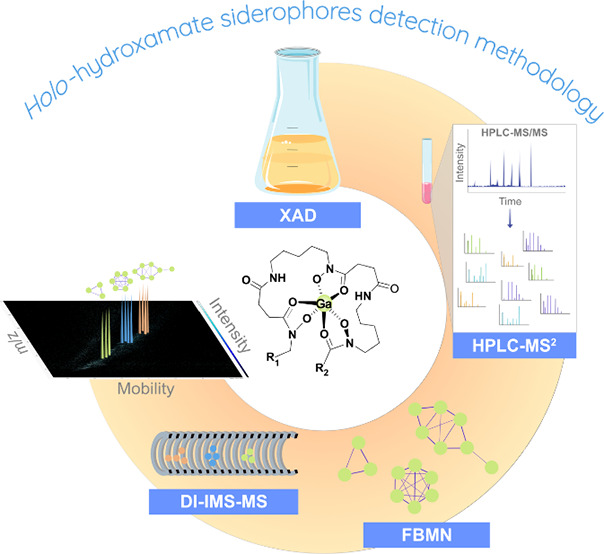

## Introduction

In response to iron deficiency, many microorganisms produce siderophores, low-molecular weight molecules with high affinity for Fe^3+^, that scavenge the surrounding iron and introduce it into the cell through specific protein receptors.^1^ Since iron is required for pathogenesis, siderophores are considered key virulence factors.^2–5^ Their scaffolds have been used for multiple biotechnologic applications including the design of novel antimicrobial strategies.^6–8^

Hydroxamate siderophores, predominantly produced by fungi and some bacterial strains,^9^ are one of the major classes of siderophores alongside catecholates/phenolates, α-hydroxycarboxylates, and mixed-type siderophores.^1^ Although most of the hydroxamate siderophores are hydrophilic, marine siderophores tend to be amphiphilic, incorporating both hydrophilic and hydrophobic moieties in their structure. The best-known hydroxamate siderophore is desferrioxamine B (DFO-B, **1**) (Figure S1A), first reported in 1960 from *Streptomyces pilosus*.^10^ Its mesylate salt, marketed as Desferal, is a FDA-approved drug used as the first-line treatment of the iron overload disease β-thalassemia.^11,12^

Despite the importance of hydroxamate siderophores, their isolation and structural elucidation remain challenging due to their low natural concentration,^13^ tendency to diffuse, and their instability, particularly in their *apo* form (unchelated). In some cases, they are produced by a single microorganism strain as a variable complex mixture of closely related analogs which complicates their purification and complete characterization.^14^ Their instability, low concentration, and rich structural diversity have been proposed as part of a defense mechanism by the producing microorganism. This strategy may help prevent their explotation by other microbes that compete for the limited iron available in the environment.^15^

Several procedures have been reported to extract or preconcentrate hydroxamate siderophores from microbial cultures prior to their separation and identification. Direct liquid–liquid extraction from the culture using organic solvents is commonly avoided due to the high polar character of these compounds.^16^ Reported extraction or prefractionation methods include the use of C-18 cartridges,^16^ hydrophobic resin CHP20P,^17^ Amberlite XAD organic polymer resins (XAD-2,^18^ XAD-4,^19,20^ XAD-7,^21^ and XAD-16 ^22^), Diaion SP 850,^23^ HP20 resin,^24^ immobilized metal ion affinity chromatography (IMAC),^25^ and most recently, titanium dioxide nanoparticles.^13^ However, most of these methods target unstable *apo-*forms,^16^ which often results in their degradation and misannotation during subsequent analysis.^26,27^

This limitation can be overcome by studying the siderophores in their *holo*-forms (metal complexes), as complexation stabilizes the siderophores and ensures their structural preservation. However, the isolation and characterization of the *holo*-forms is not common, remaining largely unexplored.^28,29^

While siderophores naturally chelate iron, they can also coordinate other metal ions, broadening their analytical and biological relevance. For instance, complexation with alternative metal ions such as Ga^3+^ has proven effective for the isolation and full characterization of unstable siderophores.^30,31^ Gallium presents a characteristic isotopic pattern (*M*_r_ = 69, 71; ratio 3:2) that facilitates the mass spectrometric detection of gallium complexes, while it enables complete NMR elucidation, which is not possible for Fe^3+^ complexes.^30,31^

Herein, we present a novel strategy for the investigation of *holo*-hydroxamate siderophores by integrating conventional extraction and isolation techniques with advanced computational tools and a unique data validation approach. This strategy, named XAD-LC/MS-FBMN-IMS, was applied to explore the siderophore metabolome (siderome) of the fish pathogen *Tenacibaculum maritimum.* Our approach not only enabled the streamlined annotation of complex metal-binding metabolite mixtures but also uncovered new insights into the pathogen’s iron uptake mechanisms. Overall, the analysis lead to the identification of 20 hydroxamate siderophores across three distinct siderophore families. Three siderophores were previously characterized and served as models to propose putative structures of the 17 new hydroxamate compounds, expanding the chemical diversity known for this economically relevant pathogen.

## Material and Methods

In this study, we established a novel workflow for the extraction and analysis of *holo*-hydroxamate siderophores. Ferrioxamine (**Fe-1**) was used as a model compound to benchmark the extraction performances of the following resins: C-18, Hydrophilic Lipophilic Balance (HLB), and Amberlite XAD. Based on the results, an optimized workflow was developed and applied to investigate siderophores produced by the fish pathogen *Tenacibaculum maritimum* LSP9.1 grown under iron-deficient conditions.

Microbial cultures were treated with GaBr_3_ to chelate and stabilize the siderophores. After centrifugation and filtration, siderophores from both the cell-free supernatant and the methanolic extract of the cell pellet were extracted using XAD-7 resin. The extracted metabolites were analyzed by HPLC-MS/MS, studied by feature-based molecular networking (FBMN), and validated via direct infusion - ion mobility spectrometry coupled to mass spectrometry (DI-IMS-MS).

A detailed description of materials and reagents, **Fe-1** synthesis and calibration curve, SPE protocols, bacterial culture conditions, *holo*-hydroxamate siderophore extraction, LC–MS/MS paramethers, FBMN anlaysis, and DI-IMS-MS validation are provided in the Supporting Information 1.

## Results and Discussion

### Optimization of *Holo-*Hydroxamate Siderophore Extraction

Effective extraction of *holo*-hydroxamate siderophores requires sorbents that fulfill two essential criteria: (1) sufficient affinity for the analyte to ensure its retention, and (2) a reversible interaction between the sorbent and the analyte to obtain high recovery percentages. Therefore, we evaluated three of the most common nonionic and nonpolar sorbents used in natural products and siderophore studies: C-18 cartridges, HLB cartridges, and Amberlite XAD resins using ferrioxamine (**Fe-1**) recoveries as a benchmark. UV–vis spectroscopy of **Fe-1** showed a characteristic maximum at 429 nm, which was used for quantification (Figure S1C).^32^ The absorption maxima of the UV–vis spectra of **1** and the Fe­(acac)_3_ salt, were also recorded to rule out possible interferences in **Fe-1** detection (Figure S1C). A calibration curve at 429 nm in the concentration range of 25–500 μM of **Fe-1** yielded an extinction coefficient of ε = 2160 ± 5.1 L mol^–1^ cm^–1^, which was consistent with previously reported coefficients for this complex (Figure S1D).^32,33^

C-18 and HLB sorbents are commonly used for the extraction and separation of organic compounds from aqueous environments. To test their affinity for *holo-*hydroxamate siderophores, 0.75 mmol of **Fe-1** were loaded onto each cartridge and eluted with a step-elution of aqueous methanol (0%, 25%, 50%, 75%, and 100%, Figure 1). Surprisingly, both sorbents exhibited strong interaction with the analyte. **Fe-1** was detected throughout all the collected fractions with maximum analyte recoveries of 18% and 19% per fraction in C-18 and HLB cartridges, respectively (Figure 1A and 1B). C-18 cartridges also failed to efficiently retain the analyte, with 28% of **Fe-1** lost during loading (Figure 1A). Contrarily, **Fe-1** was not detected in the nonretained fraction when using HLB cartridges (Figure 1B).

**Figure 1:**
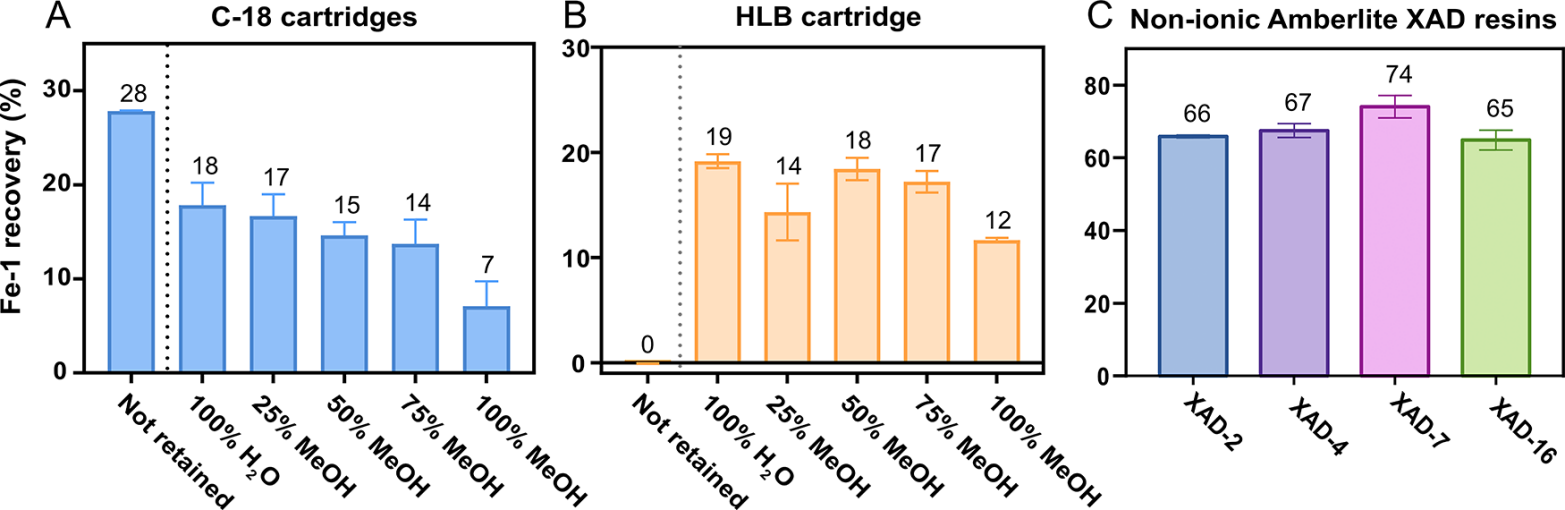
Recovery percentages of **Fe-1** from the different fractions obtained by SPE using C-18 cartridge (**A**), HLB cartridge (**B**), or nonionic Amberlite XAD resins (**C**).

Previous studies presented C-18 cartridges as the standard methodology for the isolation of organic-metal complexes in aqueous samples;^16,34–38^ however, they consistently recovered low yields, typically ranging between 10 and 30%.^16,35,37,39^ The limited recoveries of the C-18 cartridges have been attributed to the presence of free silanol groups in this stationary phase, which strongly interact with metals.^40,41^ The metallic complexes can be retained even after elution with 100% organic mobile phase, requiring the addition of acid, e.g. 0.5% HNO_3,_ to be eluted.^41^ Previous studies showing the interaction between metallic complexes and C-18 sorbents are consistent with our findings.^40,41^

HLB cartridges displayed slightly better extraction performances than C-18 and were able to efficiently retain **Fe-1** (Figure 1B). However, the cartridges still failed to concentrate the analyte into a single fraction, instead distributing **Fe-1** across multiple fractions with yields ranging from 12 to 19% (Figure 1B). This contrasts the effectiveness of HLB cartridges for the extraction of catecholate/phenolate siderophores, probably due to π-π stacking interactions facilitated by vinylpyrrolidone (hydrophilic) and divinylbenzene (lipophilic) groups that interact with the aromatic functionalities of catecholate/phenolate siderophores.^30,42,43^ In contrast, HLB cartridges appear to be less suitable for hydroxamate-type siderophores, which lack such aromatic functionalities.

Amberlite XAD resins (XAD-2, XAD-4, XAD-7, and XAD-16) were evaluated by incubating a 150 μM **Fe-1** solution with 10 mL of resin overnight, followed by MeOH desorption. The tested Amberlite XAD proved to be highly effective for the extraction of the *holo-*hydroxamate siderophore **Fe-1**, with recoveries ranging from 65 to 74% (Figure 1C). Among these resins, XAD-7 exhibited the highest recovery (74%). Hydrophobic XAD resins have been widely used for the extraction of *apo*-hydroxamate siderophores, where retention is primarily driven by hydrophobic interactions between resins and *apo*-hydroxamic siderophores.^19,44–47^ However, upon complexation with the metal core, the polarity of the siderophore increases. XAD-2, XAD-4, and XAD-16 are highly hydrophobic styrene-divinylbenzene copolymers with varying pore sizes, whereas XAD-7, a cross-linked acrylic ester, is the only “moderately polar” resin. This difference in polarity may account for the superior performance of XAD-7 in extracting *holo*-siderophores.^48^

In summary, C-18 and HLB sorbents showed high interaction and low recoveries during elution for the *holo*-hydroxamate siderophore. In contrast, XAD-7 resin provided a gentle and more effective method for extracting these compounds from aqueous samples. To the best of our knowledge, this is the first systematic comparison of extraction strategies specifically for *holo*-hydroxamate siderophores.

### Detection and Identification of *Holo*-Hydroxamate Siderophores via XAD-LC/MS-FBMN-IMS

After the optimization of the extraction method, we established a robust workflow for the accurate detection and identification of *holo*-metallophores in XAD-7 extracts. This workflow is divided in the following steps:

#### 1 Untargeted HPLC-MS/MS and Feature-Based Molecular Networking (FBMN) Analysis

Untargeted HPLC-MS/MS analysis of the XAD-7 extracts provides a comprehensive snapshot of the bacterial metabolome. Each compound exhibits a unique MS/MS fragmentation pattern, facilitating partial structural characterization and identification of structural similarities among metabolites.^49,50^ After preprocessing the precursor ions based on various features (isotope patterns, retention time, and even ion mobility separation) in MZmine, the data is submitted to the Global Natural Product Social (GNPS) platform for Feature-Based Molecular Networking (FBMN), which organizes the MS/MS spectra into networks based on spectral similarity and dereplicates ions against reported compound spectra. This approach facilitates the identification of metabolites and provides a visual representation of the entire metabolome within the analyzed sample.^49–52^

#### 2 ^69/71^Ga and ^54/56^Fe Isotopologue Screening for Potential *Holo*-Siderophores in the Generated Networks

The distinctive isotopic patterns of gallium (*M*_r_ = 69, 71; ratio 3:2) and iron (*M*_r_ = 54, 56, 57, 58; ratio 6:92:2:0.3) are easily recognizable in mass spectrometry. The addition of their salts to the sample facilitates their detection in MS by forming their corresponding metal complexes while stabilizing the analyte. There are several open-source tools available that can aid the isotopologues search, including the ‘isotope peak scanner’ function in MZmine, or the CAMERA package in R, which can annotate isotopic patterns and metal-specific adducts when combined with XCMS for peak detection and alignment.^53^

#### 3 Prediction of Molecular Formulas by SIRIUS

The chemical formulas of the detected isotopologues and their fragmentation pattern are then predicted with the open-source software SIRIUS 5.8.3.^54^ This information enables the dereplication of the data against different databases to provide putative annotations.

#### 4 Manual Ion Identity (MII) Layer for Data Simplification

The metal-binding properties of siderophores lead to the formation of multiple ion species and adducts, often with distinct retention times and MS^2^ fragmentation patterns in HPLC-MS/MS analysis. Consequently, adducts (e.g., [M + H]^+^, [M + Na]^+^, [M + K]^+^) from the same metabolite may be distributed across multiple molecular families rather than clustering together within a single network. This dispersion complicates comprehensive annotation and interpretation, hindering accurate metabolome characterization.

Several computational tools have attempted to address this issue, typically using retention time filtering and the correlation of feature intensities across spectra. These methods are poorly suited for metallophore analysis where metal-adduct forms (e.g., Fe^3+^- and Ga^3+^) may not coelute or exhibit consistent intensity correlations.^55–58^ Recently, Dorrestein and co-workers introduced ion identity molecular networking (IIMN), which relies on the presence of unbound *apo-*forms of ionophores to establish ion relationships (e.g., adducts, charge states, neutral losses) via MS^1^-based mass differences.^59^ Since most of the siderophores are present in their *holo*-forms after metal-chelation of extracts, direct application of this computational tool is challenging.

To overcome these challenges, a manual ion identity (MII) curation step was implemented to refine the network. Chemical formula predictions and MS^1^ isotopic patterns were inspected to manually identify and group ions corresponding to different adducts of the same compound (Supplementary data 2). As a result, otherwise disconnected nodes across the molecular network were linked, reducing redundancy and improving interpretability.

#### 5 Data Validation by Direct-Infusion Ion Mobility Spectrometry–Mass Spectrometry (DI-IMS-MS)

HPLC-MS/MS data used for FBMN studies generally uses reverse-phase sorbents for sample analysis. However, we observed that *holo*-hydroxamate siderophores strongly interact with these sorbents, and may form artifacts, compromising the integrity of the results. To address this, we propose the use of direct-infusion ion mobility spectrometry coupled to mass spectrometry (DI-IMS-MS) as an orthogonal layer of validation and annotation to FBMN.

IMS offers a mild, rapid (millisecond time scale), and sensitive separation of ions that provides valuable information about the composition of complex mixtures without the use of other separation techniques. Interestingly, IMS can be coupled to different detection methods such as MS that increases its value and usefulness. Ion mobility also provides specific features of each precursor ion, such as drifting time, arrival time or collision cross section (CCS) that could be potentially used as features for refining the data in FBMN. In the present study, we validated the data based on exact mass and ^54/56^Fe or ^69/71^Ga isotopologues pattern. The nodes detected via IMS were then dereplicated against different databases based on their proposed chemical formulas. To our knowledge, this study represents the first application of IMS-MS as a data validation tool for FBMN analyses.

#### 6 Putative Siderophore Structure Elucidation Based on MS/MS Fragmentation

Structural characterization of validated *holo*-siderophores was performed through careful interpretation of MS/MS fragmentation spectra. Fragmentation trees generated via SIRIUS can provide preliminary insights into substructural motifs and diagnostic neutral losses. However, the results must be considered with caution, as we observed that the program often fails to generate correct fragment annotations for metal complexes. Although NMR elucidation is usually preferred, the interaction of *holo*-hydroxamic siderophores with reverse-phase supports and complex bacterial production of highly similar analogs hinder its use. Thus, inferred structures are proposed here based on recurring fragmentation patterns, metal-retaining fragment ions, and complementary literature data.

Overall, this systematic multistep approach (Figure 2) not only serves for the identification of new siderophores but also improves our understanding of their chemical diversity and the underlying mechanisms of iron chelation.

**Figure 2:**
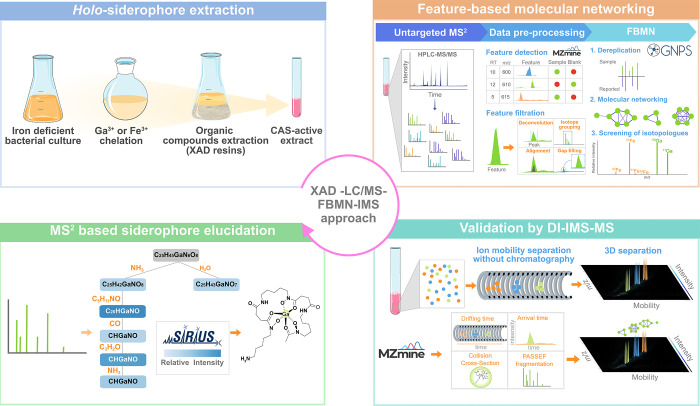
Schematic flowchart representation of the XAD-LC/MS-FBMN-IMS strategy for the analysis of *holo-*metallophores.

### Application of the XAD-LC/MS-FBMN-IMS Strategy to Unveil the Siderome of *Tenacibaculum maritimum*

The XAD-LC/MS-FBMN-IMS strategy (Figure 2) was evaluated for the detection and identification of the siderophores from *Tenacibaculum maritimum* LSP9.1, a strain isolated from a tenacibaculosis outbreak in cultured sole (*Solea senegalensis*) in Spain.

*Tenacibaculum maritimum* is the causative agent of tenacibaculosis, an ulcerative infection with lack of host specificity that severely impacts the aquaculture industry worldwide.^60,61^ Recent genomic studies of this bacterium showed the presence of iron uptake systems mediated by hydroxamate siderophores as putative virulence mechanisms.^62–64^ Notably, the detected biosynthetic gene cluster (BGC) displayed high homology for the *mbs* locus, which has been proposed to encode hydroxamic bisucaberin and desferrioxamine-like compounds.^64^ Despite the significance of *T. maritimum* as a fish pathogen in aquaculture, no studies have characterized its siderome.

To enhance the production of siderophores, *T. maritimum* LSP9.1 strain was grown in minimal medium supplemented with the chelating agent EDDHA. The presence of siderophores was confirmed by the appearance of a yellow halo around the colonies on CAS agar. However, manipulation of the culture (e.g., centrifugation) resulted in decreased CAS value, denoting unstable siderophores.

Therefore, iron-deficient cultures of *T. maritimum* LSP9.1 were treated with GaBr_3_ to stabilize the siderophores. The cell-free supernatant and cell pellet were then extracted with XAD-7 resin to afford the CAS-active extracts TMSX7M and TMPX7M, respectively. These extracts, along with the standard **Ga-1**, were subjected to HPLC-MS/MS. Acidic conditions were strictly avoided to prevent degradation and dechelation of the siderophores during separation. The limit of detection (LOD) of this analysis for **Fe-1** —used as a standard for *holo*-hydroxamate siderophores and tested over the range of 62.5 to 1.95 μg/mL— was 7.8 μg/mL. As expected, strong interaction of **Fe-1** with the RP column was observed, resulting in the detection of the complex across the entire run rather than as a distinct peak and preventing a gradual decrease in signal intensity between dilutions.

The HPLC-MS/MS data of both extracts and **Ga-1** were analyzed by FBMN (Figure S2, Tables S1 and S2). The search for ^69/71^Ga and ^54/56^Fe isotopologues revealed seven subnetworks comprising a total of 122 precursor ions putatively associated with metal-complexes (Figure 3 and Supporting Information 2). The MII layer, based on chemical formulas predicted with SIRIUS, reduced the number of chelating compounds to 45 (Supporting Information 2). Interestingly, aluminum and iron complexes were also detected, despite not being intentionally introduced, which were annotated based on isotopic patterns and exact masses (Supporting Information 2). DI-IMS-MS analysis of TM**S**X7M and TM**P**X7M extracts validated only 20 out of the 45 metal complexes detected by FBMN (Figure S3–S21). The sensitivity of DI-IMS-MS was also measured in terms of **Fe-1** concentration detecting the LOD at 7.9 pg/mL, which denotes a high sensitivity for our compounds and outcompetes the LOD displayed by HPLC-MS/MS.

**Figure 3:**
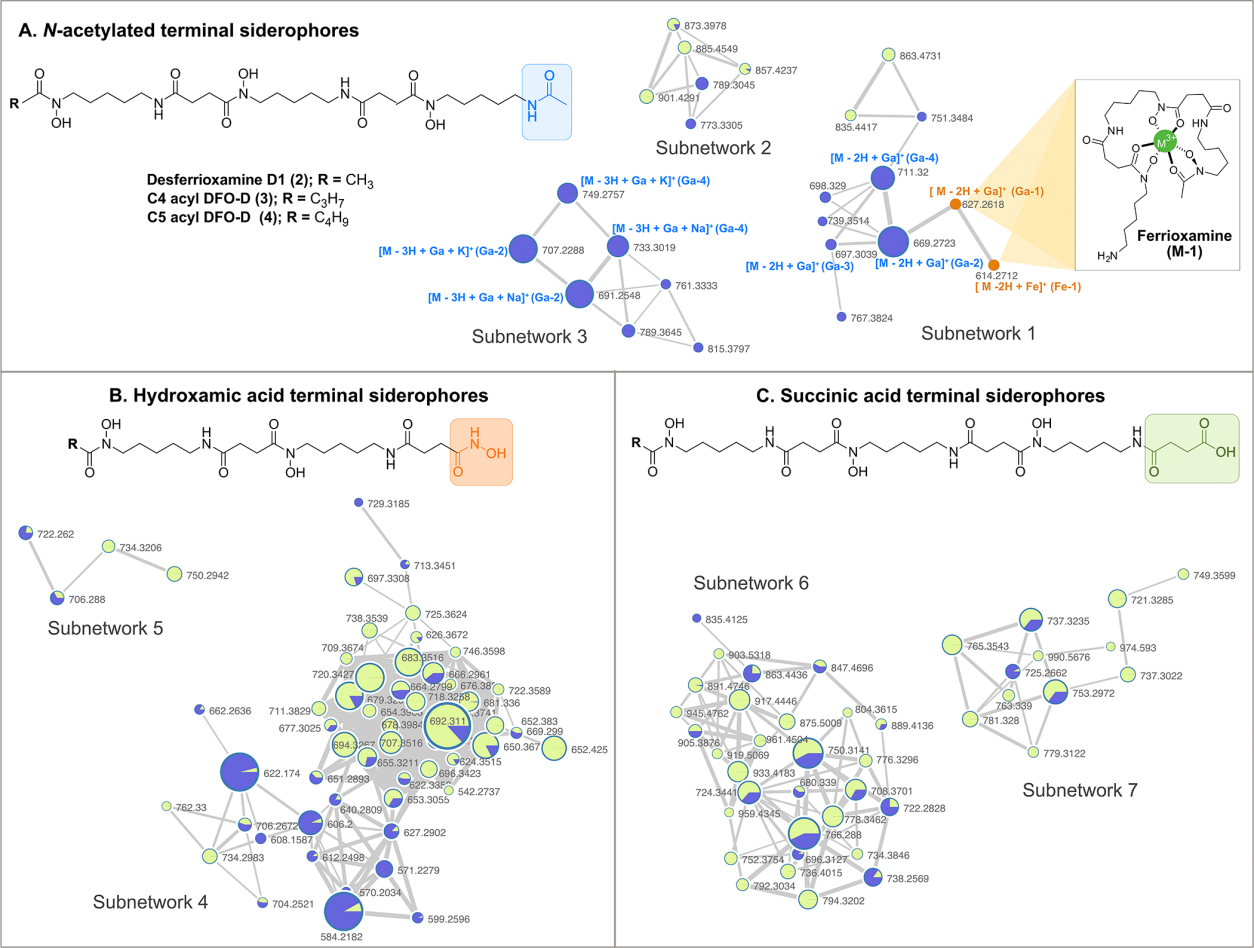
Feature-Based Molecular Networking of *Tenacibaculum maritimum* putative siderome extracted from the cell-free supernatant (blue node color) and cell pellet (green node color) and **Ga-1** and **Fe-1** used as standards (orange nodes). MS/MS analysis revealed three main families: (**A**) *N*-acetylated terminal siderophores (**M-1** signifies metal-chelated desferoxamine), (**B**) hydroxamic acid-terminal siderophores, and (**C**) *N*-succinic acid terminal siderophores. Each precursor ion is represented as a circle (node), connected to structurally similar precursors by gray connectors (edges) based on cosine similarity. Edge thickness corresponds to the cosine score, with thicker lines indicating higher structural similarity. Node radius reflects the summed precursor ion intensity, providing a semiquantitative view of siderophore abundance. Pie charts within each node indicate the relative distribution between the pellet (green) and supernatant (blue).

To elucidate their structures, we first examined the MS/MS fragmentation pattern of **Ga-1**. The MS^2^ fragmentation of this *holo*-hydroxamate siderophore can be rationalized by remote hydrogen rearrangements (RHR) (Figure S22), which confirmed the presence of the characteristic *N*-hydroxy-*N*-succinyl-cadaverine (HSC) units (Figure S22). Specifically, the fragmentation sequence begins with a neutral loss of water (fragment **a**), followed by cleavage of the *C*-terminal cadaverine unit (fragment **b**), then the loss of the *N*-terminal acetyl group (fragment **c**) and a succinyl moiety (fragment **d**). Finally, another cadaverine unit is released from the metal complex (fragment **b**), while the metal core remains intact throughout the process (Figure S22).

Analysis of the MS/MS fragmentation patterns of the 20 metal complexes revealed a common core structure with **Ga-1,** displaying *N*-hydroxy-*N*-acyl cadaverine (HAC) and HSC units, indicative of desferrioxamine-like structures. Variations included the presence of a hydroxamic acid functionality at one end or additional HSC units that enabled their classification into three distinct series (families) (Figure 3). Within each family, differences were found in the length and degree of unsaturation of the fatty acid tail in the HAC unit.

The first family of compounds is characterized by the presence of a second HSC unit, of which one is acetylated. This series is distributed across subnetworks 1, 2, and 3 (Figure 3A). Chemical formula dereplication allowed for the annotation of desferrioxamine D1 (**2**)^65^ and two previously reported siderophores from two *Streptomyces* species, C4 acyl desferrioxamine-D (C4 acyl DFO–D, **3**) and C5 acyl desferrioxamine-D (C5 acyl DFO–D, **4**) (referred in the original report as *N*-butyryldesferrioxamine B).^14,66^ Additionally, the putative structures of five new desferrioxamine D-like siderophores, compounds **5**-**9**, within this family were proposed based on their MS/MS fragmentation patterns and comparison to that of ferrioxamine D1 chelated with Ga^3+^ (**Ga-2**) (Figure 4A). Siderophores from this family displayed four characteristic neutral losses (**a**, **e**, **d**, and **b**) produced by remote hydrogen rearrangement (RHR). After losing a molecule of water (**a**), the characteristic terminal acetylated cadaverine unit is cleaved observing a neutral loss of *m*/*z* 142.1108 (**e**, calc. for C_7_H_14_N_2_O, *m*/*z* 142.1106, Δ 1.4 ppm). This cleavage is then followed by the loss of a succinyl moiety (fragment **d**, calc. for C_4_H_2_O_2_, *m*/*z* 82.0055, Δ 2.4 ppm) and a cadaverine unit (**b**, calc. for C_5_H_12_N_2_, *m*/*z* 100.1000, Δ 1.0 ppm) (Figure 4A). These fragmentations confirmed not only the presence of the two HSC units but also their structural resemblance to desferrioxamine D1 (Figure S22 and S23).

**Figure 4:**
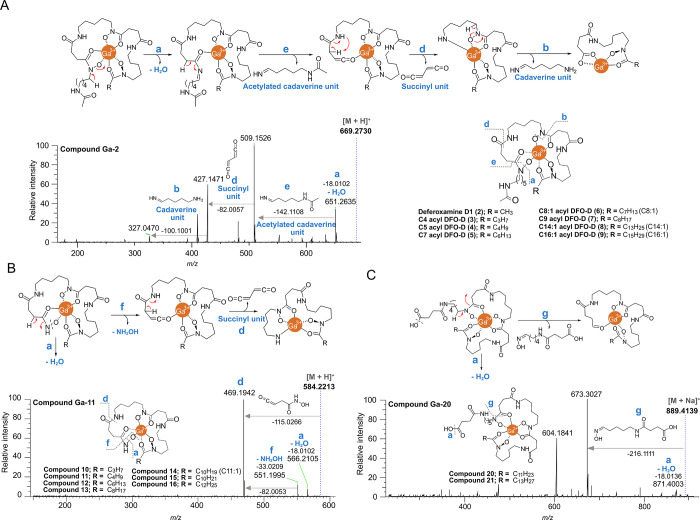
MS/MS fragmentation patterns: *N*-acetylated terminal siderophores (e.g., compound **Ga-2**) (A), hydroxamic acid-terminal siderophores (e.g., compound **Ga-11**) (B), and *N*-succinic acid terminal siderophores (e.g., compound **Ga-20**) (C).

The second familiy of compounds is characterized by the presence of a hydroxamic acid functionality at one end. This motif was previously reported in the structures of tenacibactin K, L, and M that were isolated from a coral-associated gliding bacterium of the genus *Tenacibaculum* (Figure 3B).^67^ MS/MS fragmentation analyses, explained by RHR, enabled the structural proposal of seven new putative tenacibactin-like compounds (**10**–**16**) across subnetworks 4 and 5 (Figure 4B). These tenacibactin-like siderophores displayed the loss of a water molecule (**a**) and a characteristic loss of 33.0209 Da, which matches the loss of NH_2_OH (**f**). Then, the loss of a succinyl moiety (fragment **d**, calc. for C_4_H_2_O_2_, *m*/*z* 82.0055, Δ 2.4 ppm) is also observed (Figures 4B and S24).^67^

The structures of the compounds of the third family are characterized by the presence of two HSC units and a succinyl group at one end, which resemble those of tenacibactin C isolated from a bacterium belonging to the *Tenacibaculum* genus.^68,69^ Within this group, two new structures for compounds **20** and **21** were proposed. Three additional compounds **17**–**19**, related to this family, were detected but their structures could not be predicted from the current data. MS/MS fragmentation pattern of these complexes exhibited a diagnostic neutral loss of *m*/*z* 216.1111 (**g**) proposed as the cleavage of an HSC unit (calc. for C_9_H_16_N_2_O_4_, *m*/*z* 216.1110, Δ 0.5 ppm), as depicted in Figures 4C and S25. Surprisingly, although bisucaberin B has been reported from *T. mesophilum*,^69,70^ a species closely related to *T. maritimum*, this compound was not detected in this study.

Based on the data, the exact structures of the fatty acid tail in the HAC unit at one end could not be fully established, mainly in terms of the position and configuration of the double bonds. These fatty acid chains could either be linear, mimicking those in desferrioxamine-like siderophores, or contain a terminal isopropyl group as seen in the previously reported tenacibactins.^67,68^ The amphiphilic character of the metal complexes reported here resulted in their distribution between the cell-free supernatants and the cell-pellet extracts from the cultures of *T. maritimum* LSP9.1 (Table S3). Long aliphatic chains are likely to be inserted in the bacterial membrane, avoiding diffusion in marine environments and facilitating iron transport through membrane-embedded receptors. The *N*-acetylated terminal siderophores (first family) including the three known siderophores were predominantly detected in the cell-free supernatant, while the hydroxamic acid-terminal siderophores (second family), and *N*-succinic acid terminal siderophores (third family) were detected in both the cell-free supernatant and the cell-pellet extracts from the cultures of *T. maritimum* LSP9.1 (Table S3). Some nodes were exclusive to the cell pellet; yet, the presence of siderophores in cell pellets are often overlooked. This work highlights the importance of analyzing both supernatants and cell pellets to gain a holistic understanding of the siderophore production of pathogenic bacteria. Semiquantitative analysis of the siderome based on node intensities (Figure 3) showed the hydroxamic acid-terminal tenacibactin-like siderophores as the most abundant class, followed by those terminating in a succinic acid moiety. The least abundant were the acetylated desferrioxamine-like siderophores. This distribution reflects the metabolic preference of *T. maritimum* under iron-limited conditions and underscores the structural diversity within its siderome.

In summary, we propose 17 putative structures of new *holo*-hydroxamate compounds produced by *Tenacibaculum maritimum* and detect three additional compounds whose structures remain unresolved. From this set of metallophores, desferrioxamine D1 (**2**), C4 acyl desferrioxamine-D (**3**), and C5 acyl desferrioxamine-D (**4**), were previously reported, while the remaining 17 (**5**-**21**) represent potential not previously reported new metallophores (Figure 5). To the best of our knowledge, this study represents the first comprehensive characterization of the siderome of *T. maritimum*.

**Figure 5:**
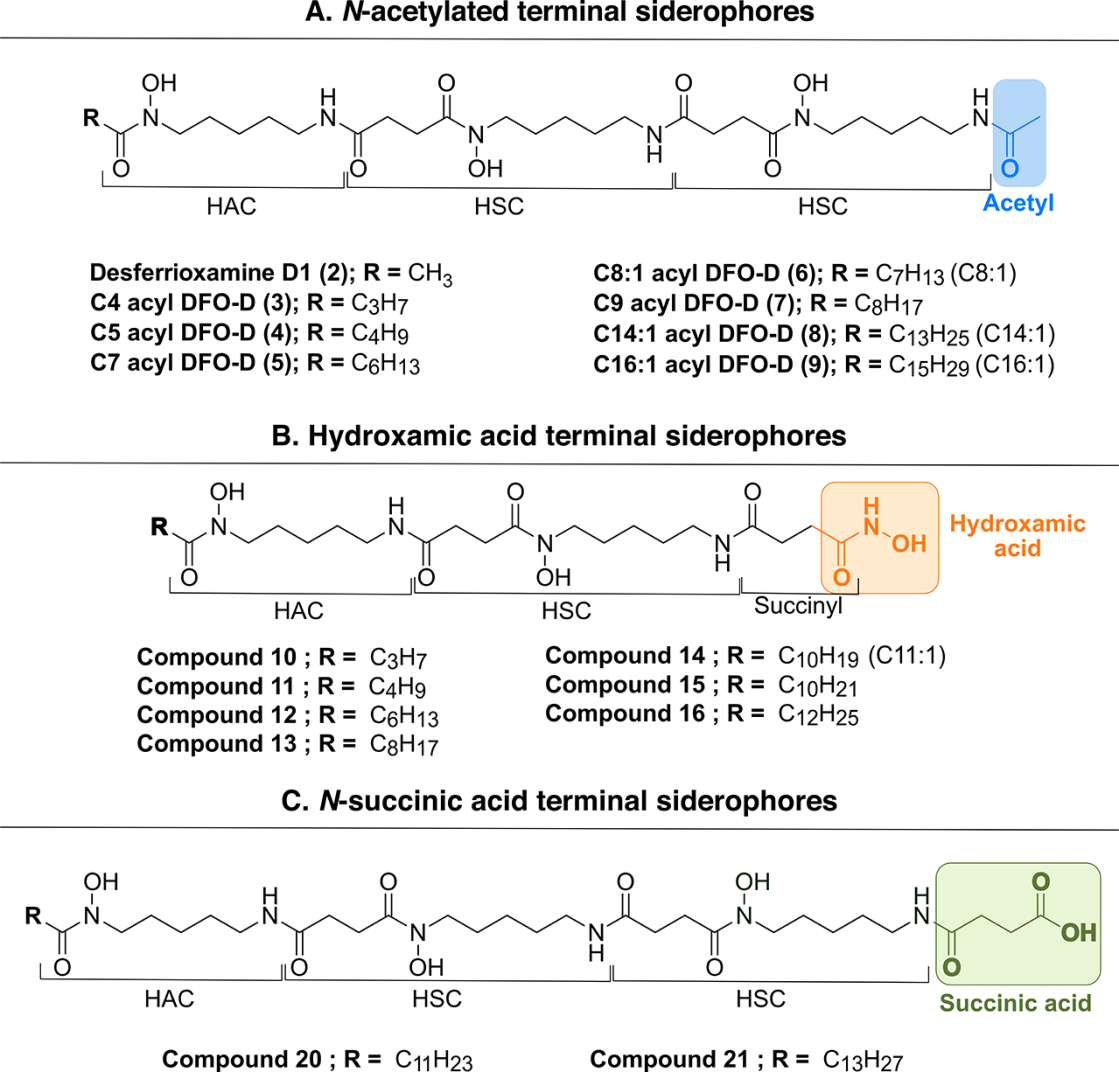
Proposed structures of the siderophores detected through our XAD-FBMN-IMS methodology corresponding to three distinct families: *N*-acetylated terminal siderophores (A), hydroxamic acid-terminal siderophores (B), and *N*-succinic acid terminal siderophores (C).

### Strengths and Limitations of the XAD-LC/MS-FBMN-IMS Strategy

This study utilizes direct-infusion ion mobility spectrometry coupled with mass spectrometry (DI-IMS-MS) as a novel data validation layer within a Feature-Based Molecular Networking (FBMN) workflow. While IMS provides distinct advantages, due to its versatility in data validation and separation, it also has limitations when applied to complex mixtures.

IMS offers a mild, rapid (millisecond time scale), and sensitive separation of ions that provides valuable information about the composition of complex mixtures without the use of other separation techniques.^71,72^ It is particularly effective for resolving complex mixtures of reverse-phase sensitive analytes and isomeric or isobaric compounds - such as isomers with different double bond positions, like compounds **6**, **8**, and **9** - that are often challenging to distinguish using liquid chromatography alone. The additional molecular descriptors generated by IMS (e.g., drifting time, arrival time or collision cross section) also improve the detection and filtering of the metabolites, leading to cleaner and more robust data in FBMN. However, availability of databases that incorporate these parameters are still limited.

DI-IMS also displays some drawbacks related to low separation resolution and low IMS-MS sensitivity along with the lack of metallophores standards and software limitations. DI-IMS resolving power is lower than that of liquid chromatography, commonly resulting in the coelution of analytes. Therefore, ion mobility separation can sometimes negatively affect the sensitivity of mass detection, which could in turn influence the finding of low abundance analytes. For this reason, optimization of IMS parameters is crucial for the analysis.

The lack of software packages that incorporate DI-IMS-MS data remains a major bottleneck of this strategy. Although ion mobility data has recently been incorporated into open source processing tools like MZmine, current software does not support the analysis of data obtained via direct infusion. Similarly, new software tools are needed for the ion identity of metal complexes within the FBMN workflow that do not rely on the presence of *apo*-forms in the mixture. Due to these limitations, we ultimately performed this layer of analysis manually, highlighting the need for further software development.

The analysis of siderophores and other metallophores presents unique challenges due to the absence of commercial standards and specialized software tools for their analysis and structural elucidation. These limitations can make reliable identification difficult, especially when dealing with new molecules. However, advancements in computational tools and community-driven database development hold promise for improving the identification and characterization of these important biomolecules.

## Conclusions

A novel, robust, and high-sensitivity strategy is presented for comprehensive characterization of *holo*-hydroxamate siderophores and metallophores. By integrating XAD-based extraction with Feature-Based Molecular Networking (FBMN) and data validation through direct-infusion ion mobility spectrometry-mass spectrometry (DI-IMS-MS), we overcame limitations of traditional chromatographic methods, particularly for C-18 sensitive analytes.

This innovative strategy was successfully applied to detect siderophores produced by *Tenacibaculum maritimum*, a pathogenic marine bacterium of significant ecological and aquacultural concern. Using this methodology, we were able to propose the structures of 20 hydroxamate siderophores, of which 17 had not been reported. These compounds were distributed across both the cell-free supernatant and cell-pellet extracts. This work represents the first detailed siderome characterization of *T. maritimum*, providing key insights into the iron acquisition strategies of this marine pathogenic bacterium as one of its key virulence factors.

While IMS does not replace chromatographic separation in terms of resolution, its application as a complementary validation layer in FBMN workflows, particularly for hydrophilic, reverse-phase sensitive, or isomeric compounds, was thoroughly demonstrated. This work deepens the research on the extraction and structural elucidation of hydroxamate-type siderophores. At the same time, it lays the groundwork for future research into the development of improved computational tools that integrate DI-IMS data into FBMN workflows, providing novel solutions for the analysis of metal complexes. Overall, this workflow significantly improves siderophore research by providing a versatile, reproducible, and comprehensive strategy. Its application in the analysis of siderophores from microorganisms will increase our understanding of microbial metal acquisition and their role in bacterial or fungal virulence, paving the way for potential applications in medicine, agriculture, and environmental remediation. Furthermore, we believe the XAD-LC/MS-FBMN-IMS strategy is also suited for the analysis of other metallophores or analytes exhibiting strong reverse-phase interactions.

## Supplementary Material





## Data Availability

All MS data are publicly available via MassIVE (https://massive.ucsd.edu) under the accession number MSV000098037.

## Notes

Data analysis, including the chemical formulas, adducts, and exact masses found in subnetworks 1 to 7 (XLSX)
Detailed description of the methodology used for this work; data-dependent acquisition parameters; MZmine parameters; Global molecular network; DI-IMS-MS extracted mobilograms for all the metallophores and their corresponding MS spectra; MS/MS scatter plots of ferrioxamine B and the metallophores found in *Tenacibaculum maritimum* (PDF)
