# Supplementary material for: Novel XAD-LC/MS-FBMN-IMS Strategy for Screening Holo-Hydroxamate Siderophores: Siderome Analysis of the Pathogenic Bacterium Tenacibaculum maritimum
Source: Anal Chem. 2025 Jun 16;97(25):13376–85. doi: 10.1021/acs.analchem.5c01687 (PMC12224167; doi:10.1021/acs.analchem.5c01687)
Supplement: Supplementary file 2 [file ac5c01687_si_002.pdf]

Supporting Information 1

Novel XAD-LC/MS-FBMN-IMS Strategy for Screening *Holo*-Hydroxamate Siderophores: Siderome Analysis of the Pathogenic Bacterium *Tenacibaculum maritimum*

Lucía Ageitos,[1] Larissa Buedenbender,[1] M. Pilar Escribano,[2] Abel Mateo Forero,[1] Beatriz Santos,[2] Miguel Balado,[2] Manuel L. Lemos,[2] Beatriz Magariños,[2] Jaime Rodríguez,[1*] Carlos Jiménez.[1*]

[1] Departmento de Química, Facultad de Ciencias and CICA - Centro Interdisciplinar de Química e Bioloxía, Universidade da Coruña, A Coruña 15071, Spain.

[2] Department of Microbiology and Parasitology, Aquatic One Health Research Center (iARCUS), Universidade de Santiago de Compostela, Santiago de Compostela, Spain.

*Corresponding author

Index

1. Methodology S4

Material S4

Bacterial and growth conditions S4

Detection of siderophores by Chrome Azurol S (CAS) assay in agar plates S4

Ferrioxamine calibration curve S4

Figure S1: (A) Synthesis of ferrioxamine (Fe-1) by treatment of desferrioxamine B (DFO-B, 1) with $Fe(acac)_3$, (B) (+)-HR-ESIMS of Fe-1, (C) UV absorption spectra of DFO-B (1), ferrioxamine (Fe-1), and $Fe(acac)_3$, and (D) calibration curve of ferrioxamine (Fe-1) between concentrations of measured at 429 nm. S5

Ferrioxamine extraction optimization with XAD resins S6

Ferrioxamine extraction optimization with SPE cartridges S6

Tenacibaculum maritimum supernatant siderophore isolation S6

Tenacibaculum maritimum pellet siderophore isolation S6

Untargeted HPLC-MS/MS analysis of TMSX7M and TMPX7M S7

Table S1: MS and DDA acquisition parameters for TMSX7M, TMPX7M, and ferrioxamine (chelated with gallium, Ga-1) analyses S8

Feature-based molecular networking S9

Table S2: MZmine parameters for TMSX7M, TMPX7M, and Ga-1 analyses S10

Figure S2: Global molecular network from Tenacibaculum maritimum LSP9.1. Nodes in green belong to the cell-pellet, while nodes in blue were extracted from the cell-free supernatant. In orange are represented the nodes corresponding the ferrioxamine D1, added as a standard. . S11

Manual ion identity (MII) layer for data simplification S12

Direct infusion ion mobility mass spectrometry and LOD determination S12

2. Direct-injection ion mobility spectrometry validation S14

Figure S3: DI-IMS-MS validation of desferrioxamine D1 (2): (A) Ion mobilogram showing a mobility of $1/K_0$ [V·s/cm^2] = 1.152. (B) (+)-HRMS spectrum of the [M-3H+Ga+Na]$^+$ ion at *m/z* 691.2565. S14

Figure S4: DI-IMS-MS validation of C4 acyl DFO-D (3): (A) Ion mobilogram showing a mobility of $1/K_0$ [V·s/cm^2] = 1.186. (B) (+)-HRMS spectrum of the [M-3H+Ga+Na]* ion at *m/z* 719.2890..... S15

Figure S5: DI-IMS-MS validation of C5 acyl DFO-D (4): (A) Ion mobilogram showing a mobility of $1/K_0$ [V·s/cm^2] = 1.210. (B) (+)-HRMS spectrum of the [M-3H+Ga+Na]$^+$ ion at *m/z* 733.3033. .... S16

Figure S6: DI-IMS-MS validation of C7 acyl DFO-D (5): (A) Ion mobilogram showing a mobility of $1/K_0$ [V·s/cm^2] = 1.243. (B) (+)-HRMS spectrum of the [M-3H+Ga+Na]$^+$ ion at *m/z* 761.3351. .... S17

Figure S7: DI-IMS-MS validation of C8:1 acyl DFO-D (6): (A) Ion mobilogram showing a mobility of $1/K_0$ [V·s/cm^2] = 1.215 (B) (+)-HRMS spectrum of the [M-3H+Ga+Na]$^+$ ion at *m/z* 773.3339. ..... S18

Figure S7: DI-IMS-MS validation of C9 acyl DFO-D (7): (A) Ion mobilogram showing a mobility of $1/K_0$ [V·s/cm^2] = 1.285. (B) (+)-HRMS spectrum of the [M-3H+Ga+Na]$^+$ ion at *m/z* 789.3648. .... S19

Figure S8: DI-IMS-MS validation of C14:1 acyl DFO-D (8): (A) Ion mobilogram showing a mobility of $1/K_0$ [V·s/cm^2] = 1.341. (B) (+)-HRMS spectrum of the [M-3H+Ga+Na]$^+$ ion at *m/z* 857.4269. .... S20

Figure S9: DI-IMS-MS validation of C16:1 acyl DFO-D (9): (A) Ion mobilogram showing a mobility of $1/K_0$ [V·s/cm^2] = 1.379. (B) (+)-HRMS spectrum of the [M-3H+Ga+Na]$^+$ ion at *m/z* 885.4588. .... S21

Figure S10: DI-IMS-MS validation of compound 10: (A) Ion mobilogram showing a mobility of $1/K_0$ [V·s/cm^2] = 1.152. (B) (+)-HRMS spectrum of the [M-3H+Ga+Na]$^+$ ion at *m/z* 592.1878. .... S22

Figure S11: DI-IMS-MS validation of compound 11: (A) Ion mobilogram showing a mobility of $1/K_0$ [V·s/cm^2] = 1.062. (B) (+)-HRMS spectrum of the [M-3H+Ga+Na]$^+$ ion at *m/z* 606.2034. .... S23

Figure S12: DI-IMS-MS validation of compound 12: (A) Ion mobilogram showing a mobility of $1/K_0$ [V·s/cm^2] = 1.100. (B) (+)-HRMS spectrum of the [M-3H+Ga+Na]$^+$ ion at *m/z* 634.2351. .... S24

Figure S13: DI-IMS-MS validation of compound 13: (A) Ion mobilogram showing a mobility of $1/K_0$ [V·s/cm^2] = 1.153. (B) (+)-HRMS spectrum of the [M-3H+Ga+Na]$^+$ ion at *m/z* 662.2678. .... S25

Figure S14: DI-IMS-MS validation of compound 14: (A) Ion mobilogram showing a mobility of $1/K_0$ [V·s/cm^2] = 1.173. (B) (+)-HRMS spectrum of the [M-3H+Ga+Na]$^+$ ion at *m/z* 688.2817. .... S26

Figure S15: DI-IMS-MS validation of compound 15: (A) Ion mobilogram showing a mobility of $1/K_0$ [V·s/cm^2] = 1.191. (B) (+)-HRMS spectrum of the [M-3H+Ga+Na]$^+$ ion at *m/z* 690.2976. .... S27

Figure S16: DI-IMS-MS validation of compound 16: (A) Ion mobilogram showing a mobility of $1/K_0$ [V·s/cm^2] = 1.233. (B) (+)-HRMS spectrum of the [M-3H+Ga+Na]$^+$ ion at *m/z* 718.3292. .... S28

Figure S17: DI-IMS-MS validation of compound 17: (A) Ion mobilogram showing a mobility of $1/K_0$ [V·s/cm^2] = 1.205. (B) (+)-HRMS spectrum of the [M-3H+Ga+Na]$^+$ ion at *m/z* 722.2862. .... S29

Figure S18: DI-IMS-MS validation of compound 18: (A) Ion mobilogram showing a mobility of $1/K_0$ [V·s/cm^2] = 1.245. (B) (+)-HRMS spectrum of the [M-3H+Ga+Na]$^+$ ion at *m/z* 750.3180. .... S30

Figure S19: DI-IMS-MS validation of compound 19: (A) Ion mobilogram showing a mobility of $1/K_0$ [V·s/cm^2] = 1.2861. (B) (+)-HRMS spectrum of the [M-3H+Ga+Na]$^+$ ion at *m/z* 778.3494. .... S31

Figure S20: DI-IMS-MS validation of compound 20: (A) Ion mobilogram showing a mobility of $1/K_0$ [V·s/cm^2] = 1.371. (B) (+)-HRMS spectrum of the [M-3H+Ga+Na]$^+$ ion at *m/z* 889.4182. .... S32

Figure S21: DI-IMS-MS validation of compound 21: (A) Ion mobilogram showing a mobility of $1/K_0$ [V·s/cm^2] = 1.152. (B) (+)-HRMS spectrum of the [M-3H+Ga+Na]$^+$ ion at *m/z* 917.4507. .... S33

3. MS/MS fragmentation patterns S34

Figure S22: MS/MS fragmentation pattern of desferrioxamine B (1) chelated with gallium (Ga-1): (A) Proposed fragmentation pathway of Ga-1 based on remote hydrogen rearrangements, (B) MS/MS spectrum from the [M + H]$^+$ ion of Ga-1, and (C) proposed fragmentation tree by SIRIUS 5.8.3. .... S34

Figure S23: MS/MS fragmentation pattern from the [M - 2H + Ga]$^+$ ions of the metallophores A) C4 acyl DFO-D (3), B) C5 acyl DFO-D (4), C) C7 acyl DFO-D (5), D) C8:1 acyl DFO-D (6), E) C9 acyl DFO-D (7), F) C14:1acyl DFO-D (6), and G) C16:1 acyl DFO-D (9). S35

Figure S24: MS/MS fragmentation pattern from the [M - 2H + Ga]$^+$ ions of the metallophores: A) compound 10, B) compound 12, C) compound 13, D) compound 14, E) compound 15, and F) compound 16. .... S36

Figure S25: MS/MS fragmentation pattern from the [M - 3H + Ga + Na]$^+$ ions of the metallophores: A) compound 17, B) compound 18, C) compound 19, and D) compound 21. .... S37

Table S3: Chemical formulae, feature-based molecular networking, and DI-IMS-MS data of the metallophores found in the pellet and supernatant of *Tenacibaculum maritimum* LSP9.1 ...... S38

1. Methodology

Material

Desferrioxamine B mesylate salt, ethylenediaminetetraacetic acid (EDTA), ethylenediamine-N,N'-bis(2-hydroxyphenylacetic acid) (EDDHA), and XAD resins were purchased from Sigma-Aldrich. HLB cartridges were purchased from Waters and C18 cartridges from Thermo Fisher. The solvents used in the HPLC-HRMS and HPLC analyses were LC/MS or LC grade, respectively. MeOH and EtOAc employed for the fractionation were reagent grade (Fisher®). Milli-Q water (MQ-H_2O) was obtained from a Direct-Q ® system (Merck). PTFE syringe filters were purchased from Macherey-Nagel. Discovery HS F5 column (100 mm × 4.6 mm, 5 μm, Supelco) used for the HPLC-MS/MS analyses was obtained from Sigma-Aldrich.

HRMS, HPLC-HRMS, and HPLC-MS/MS data were acquired on a LTQ-Orbitrap Discovery mass spectrometer coupled to an Accela HPLC (Thermo Scientific).

Bacterial and growth conditions

Tenacibaculum maritimum LSP9.1 was grown on *Flexibacter maritimus* medium (FMM) agar plates for 48 h at 25 °C. Pre-inoculums were prepared by adding one colony in 400 mL of FMM broth and shaken at 25 °C for 48 h. Once an OD_{600}= 0.5 was achieved, the pre-inoculum was added to 2 L of M9 media supplemented with 0.4% casamino acids (CM9) with ethylenediamine-N,N'-bis(2-hydroxyphenylacetic acid (EDDHA) at a working concentration of 20 μM. After 48 h at 25 °C, the culture was chelated with $GaBr_3$ (10 μM) and incubated at 25 °C for 24 h to stabilize the potential siderophores in the medium before the filtration of the culture. Subsequently, the culture was pelleted at 6000 rpm for 30 min at 4 °C (Avanti TM J25) and continuously filtrated through 0.45 μm filters (prep/scale®-TFF cartridge, Millipore®). The cell-free supernatants were collected and stored at -20 °C until their extraction. The cell-pellets were suspended on 40 mL MeOH and stored at -20 °C.

Detection of siderophores by Chrome Azurol S (CAS) assay in agar plates

Bacterial solutions at an adjusted OD_{600} of 0.8 were submitted to the liquid chrome azurol-S (CAS) assay following the method described by Schwyn & Neilands (1987). For the assay, equal volumes of cell-free supernatant and CAS reagent were combined and incubated at room temperature for 15 minutes. The absorbance at 630 nm (A630) was then recorded using a UV-VIS spectrophotometer (Hitachi).

Ferrioxamine calibration curve

A solution of desferrioxamine B mesylate salt [DFO-B (**1**), 46.6 mg, 0.07 mmol, 1 equiv.] in MQ-H_2O (2.5 mL) was treated with $Fe(acac)_3$ (26.8 mg, 0.07 mmol, 1 equiv.) for 24 h under stirring at room temperature (Figure S1A). The reaction mixture was then washed with EtOAc (3 x 10 mL), concentrated under reduced pressure conditions, and freeze-dried to afford 53 mg of a red solid. This residue was purified via Sephadex LH20 (65 mL) using MeOH as eluent to afford the final product as a red solid in 82% yield (44.6 mg), identified as ferrioxamine (**Fe-1**) by (+)-HR-ESIMS. The formation of **Fe-1** was confirmed upon its (+)-HR-ESIMS, displaying the $[M + H]^+$ ion at *m/z* 614.2726 (calcd. for $C_{25}H_{46}FeN_6O_8^+$, *m/z* 614.2715, Δ 1.8 ppm) as the base peak and the characteristic isotopic pattern of Fe (*Mr* = 54, 56, 57, 58; ratio 6:92:2:0.3) (Figure S1B).

Desferrioxamine (**1**), ferrioxamine (**Fe-1**), and $Fe(acac)_3$ at a concentration of 150 μM in MQ-H_2O were spectrophotometrically studied in the range 200-800 nm using a Jasco V-650 spectrometer and a quartz cuvette of 1 cm optical path (Figure S1C). Ferrioxamine (**Fe-1**)

showed a characteristic maximum at 429 nm in the UV spectrum, which was selected for the creation of a calibration curve.

A stock solution of 1124.67 µM was obtained by dissolving 6.9 mg of ferrioxamine (**Fe-1**) in 10 mL of MQ-H_2O. After measuring the blank with MQ-H_2O, the calibration curve was performed by sequential additions of the stock solution to obtain the following concentrations: 0, 25, 50, 100, 150, 200, 250, 350, and 500 µM. The experiment was performed with three independent experimental replicates and three instrumental replicates. The obtained calibration curve was: y= 0.002160x + 0.003508 with a R^2=0.999 (Figure S1C).

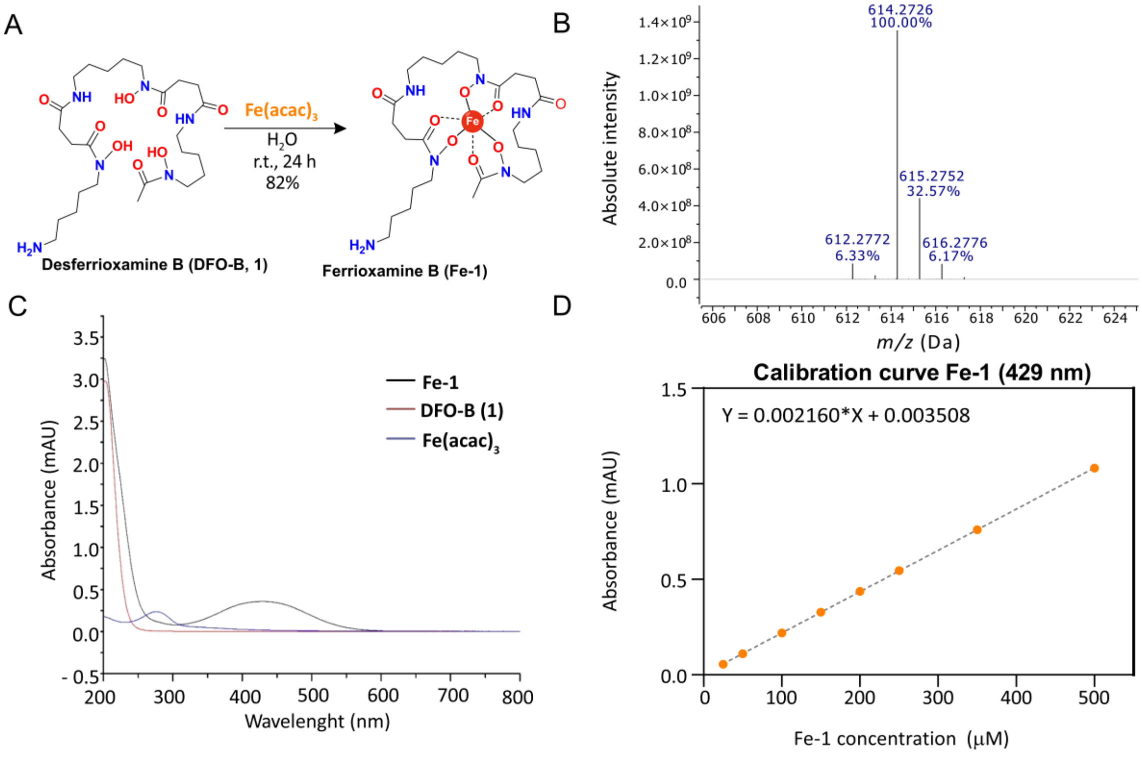

Figure S1: (A) Synthesis of ferrioxamine (**Fe-1**) by treatment of desferrioxamine B (DFO-B, **1**) with Fe(acac)$_3$, (B) (+)-HR-ESIMS of **Fe-1**, (C) UV absorption spectra of DFO-B (**1**), ferrioxamine (**Fe-1**), and Fe(acac)$_3$, and (D) calibration curve of ferrioxamine (**Fe-1**) between concentrations of measured at 429 nm.

Ferrioxamine extraction optimization with XAD resins

XAD-2, XAD-4, XAD-7 and XAD-16 resins were washed with MeOH for 24 hours at room temperature and thoroughly rinsed with MQ-H_2O (7 cycles) immediately before their use.

Three batches of 5 mL of pre-washed XAD-7 resin were treated with three different solutions of ferrioxamine (**Fe-1**) at 150 µM, 250 µM, and 450 µM in 5 mL and shook in a platform shaker (80 rpm) overnight at 4 ºC. The non-retained solution was then removed, and the organic compounds were desorbed with 3 bed volumes (BV) of MeOH (15 mL). The non-retained and desorbed fractions were lyophilized and studied at 429 nm to calculate the percentage of ferrioxamine (**Fe-1**) in both fractions.

Since the best results were obtained at 150 µm, the experiment was repeated using 10 mL of XAD-7 and 30 mL of MeOH. As the percentage of recovery increased, the same conditions were repeated with the rest of the resins (XAD-2, XAD-4, and XAD-16) to compare their affinity for ferrioxamine (**Fe-1**). Three independent replicates were studied for each resin.

Ferrioxamine extraction optimization with SPE cartridges

C18 (200 mg) and HLB cartridges (200 mg) were pre-conditioned with the following mixtures of MQ-H_2O:MeOH (A:B): 0:1, 1:1, and 1:0. Then, 0.75 mmol of ferrioxamine (**Fe-1**) in 1 mL of MQ-H_2O were loaded onto each cartridge, collecting the filtrate to analyzed the not-retained (N.R.) portion of analyte. The sample was then fractionated with 0, 25, 50, 75 and 100 % of MeOH in MQ-H_2O. The different fractions were freeze-dried to afford residues that were dissolved in 2 mL of MQ-H_2O and analyzed by UV-Vis. This experiment was performed in three independent triplicates and three instrument replicates.

Tenacibaculum maritimum supernatant siderophore isolation

Positive CAS assay cell-free supernatants (1L) from the gallium-chelated cultures were collected and washed with EtOAc (2 x 500 mL) to eliminate nonpolar compounds. The remanent content of EtOAc in the aqueous fraction was removed under reduced pressure. The aqueous fraction was then absorbed onto the Amberlite non-ionic XAD-7 resin (1L) overnight at 4 ºC under stirring in a waving platform shaker (80 rpm). The resin was loaded in an open glass column and washed with H_2O. After removing all the salts, measured by $AgNO_3$ precipitation assay, the organic content was desorbed with one column volume (CV) of MeOH. The resultant extract was concentrated under reduced pressure, affording an aqueous solution that was treated with $Ga(acac)_3$ (0.1 mM) for 5 minutes. The suspension was allowed to stir for 10 minutes at room temperature and then it was incubated at 4 °C overnight. The gallium-chelated extract was lyophilized to afford a residue named TMSX7M. To ensure reproducibility, three biological replicates and three experimental replicates were used for this analysis. All the materials needed for the extraction were cleaned with 1M HCl overnight to remove any iron traces and subsequently rinsed with MQ-H_2O. The XAD-7 resin was washed with MeOH (1CV), rinsed with an EDTA solution (1CV) to remove iron, and washed thoroughly with deionized water (7CV) prior to its use.

Tenacibaculum maritimum pellet siderophore isolation

The cell pellet from the gallium pre-chelated *T. maritimum* culture was extracted twice with MeOH (30 mL) and filtered through a 0.22 µm PTFE filter. The resultant methanolic extracts were combined and treated with 40 mg of $Ga(acac)_3$ over 5 min. The suspension was allowed to stir for 10 min at room temperature and then it was incubated at 4 °C overnight. The resultant solution was concentrated *in vacuo* to afford a residue that was dissolved in MQ-H_2O (50 mL). The aqueous solution was adsorbed on Amberlite non-ionic XAD-7 resin

(70 mL) overnight at 4 ºC under stirring on a waving platform shaker (80 rpm). After removing the non-retained solution, the resin was thoroughly washed with MilliQ water until no salts were observed in the $AgNO_3$ precipitation assay. The organic content was then desorbed with 1CV of MeOH and concentrated under reduced pressure followed by lyophilization to afford a brown residue named TMPX7M. To ensure reproducibility, three biological replicates and three experimental replicates were used for this analysis. All the materials needed for the extraction were cleaned with 1M HCl overnight to remove any iron traces and subsequently rinsed with MilliQ-H_2O. The XAD-7 resin was washed with MeOH (1CV), rinsed with an EDTA solution (1CV) to remove iron, and washed thoroughly with deionized water (7CV) prior to its use.

Untargeted HPLC-MS/MS analysis of TMSX7M and TMPX7M

HPLC-HRMS profiles of TMSX7M, TMPX7M, and a solvent blank were acquired on a HPLC Accela coupled to an LQT-Orbitrap Discovery mass spectrometer (Thermo Fisher Scientific) and a PDA detector. Samples were prepared at a concentration of 1 mg mL^{-1} in H_2O:MeCN (9:1) and filtered through 0.2 µm PTFE syringe filters (Macherey-Nagel, Germany). A Discovery HS F5 column (100 mm × 4.6 mm, 5 µm; Supelco) at 30 ºC was used for chromatographic separation with a flow rate of 0.5 mL min^{-1} (Solvent A: H_2O, Solvent B: MeCN). The HPLC elution protocol was set as follows: an isocratic step of 2 min at 20%, 18 min from 20 to 70 %, 5 min from 70 to 100 %, a 5-min isocratic step at 100 %, 1 min from 100 to 10%, and a final 4-min isocratic step at 10% of B. Mass spectra were acquired in positive mode, with resolution set to 30.000 in the range of *m/z* 150–1500. MS parameters were set as follows: spray voltage of 3.90 kV, capillary temperature of 350 °C, sheath gas rate of 80 units N_2 (ca. 800 mL/min), and auxiliary gas rate of 20 units N_2 (ca. 200 mL/min). To perform molecular networking, data were recorded in data-dependent acquisition (DDA) mode, in which the five most intense ions in the full-scan mass spectrum with a minimum signal threshold of 10^5 counts were subjected to high-resolution tandem mass spectrometry analysis via collision-induced dissociation (CID) fragmentation. CID fragmentation was set with an isolation width of 3.00 Da, normalized collision energy of 35, an activation Q of 0.250 units, and an activation time of 30 ms. Dynamic exclusion was enabled to 5 repeat counts, 10 s of repeat duration, list size of 50, and exclusion duration of 20 s. The exclusion mass width was set to -0.01 and +2.1 Da to exclude MS2 fragmentation of isotope peaks (e.g. ^{71}Ga).

Table S1: MS and DDA acquisition parameters for TMSX7M, TMPX7M, and ferrioxamine (chelated with gallium, **Ga-1**) analyses

Parameter	Value
**MS parameters**	
Spray voltage	3.90 kV
Capillary temperature	350 °C
Sheat gas	80 units N_2 (ca. 800 mL min^{-1})
Auxiliary gas rate	20 units N_2 (ca. 200 mL min^{-1})
Noise Level	5.10^4
**Data Dependent Acquisition (DDA)**	Topmost intense five ions
Maximum ion injection time MS^1	100 ms
Maximum ion injection time MS^2	100 ms
CID isolation width	3
CID normalized collision energy	35.0 eV
Activation Q	0.250
Activation time	30 ms
**Dynamic exclusion**	
Repeat count	5
Repeat duration	10 s
Exclusion list size	50
Exclusion duration	20 s
Exclusion mass width	-0.01 to +2.1

Feature-based molecular networking

The raw data files were converted to mzXML format using ProteoWizard and the peak-picking filter and then imported via WinSCP to MZmine v3.3.0 for preprocessing. Mass detection was set to a noise level of 5.10^4 for the MS^1 level and 5.10^3 for the MS^2 level. Chromatograms were built based on a minimum peak height of 5.10^4 counts, a minimum group size of 4 scans, and a *m/z* tolerance of 0.002 Da or 10 ppm. Chromatogram deconvolution was performed using the Local Minimum Search algorithm (chromatographic threshold: 0.9, minimum RT range: 0.1 min, minimum relative height: 0%, minimum absolute height: 5.10^4, top/edge peak ratio: 1.5, peak duration 0.01-10.00 min, and minimum number of data points: 3). ^{13}C-isotope peaks were removed with the isotope peak grouper algorithm with *m/z* tolerance of 0.002 (or 10 ppm) and RT tolerance of 0.1 min. The data were filtered to only retain ions with MS^2 spectra. All samples were combined in a peak list using the join aligner algorithm (75% weight for *m/z*, and 25% weight for RT) and those ions detected in the solvent blank were removed from the peak list (minimum number of scans detected on the blank set to 3). Subsequently, gap-filling and duplicate filtering were performed. Finally, the data were exported as .csv and .mgf files and uploaded to the GNPS platform for feature-based molecular networking analysis and to SIRIUS 5.8.3.

A molecular network was created with the Feature-Based Molecular Networking (FBMN) workflow on GNPS2. MS/MS spectra were window-filtered by choosing only the top 6 fragment ions in the +/- 50 Da window throughout the spectrum. The precursor ion mass tolerance was set to 0.02 Da and the MS/MS fragment ion tolerance to 0.02 Da. A molecular network was then created where edges were filtered to have a cosine score above 0.7 and more than 5 matched peaks. Further, edges between two nodes were kept in the network if and only if each of the nodes appeared in each other's respective top 40 most similar nodes. Finally, the maximum size of a molecular family was set to 100, and the lowest-scoring edges were removed from molecular families until the molecular family size was below this threshold. The spectra in the network were then searched against GNPS spectral libraries. The library spectra were filtered in the same manner as the input data. All matches kept between network spectra and library spectra were required to have a score above 0.6 and at least 6 matched peaks. The dereplicator was used to annotate MS/MS spectra. The molecular networks were visualized using Cytoscape software. Link to this work GNPS data:

https://gnps2.org/status?task=b8daf33dbe164a669ff9cc489000b221

Table S2: MZmine parameters for TMSX7M, TMPX7M, and **Ga-1** analyses

Parameter	Value
**Mass detection**	
RT range	0-35 min
MS Level	1
Mass detector	Centroid
Noise Level	5.10^4
**Mass detection**	
RT range	0-35min
MS Level	2
Mass detector	Centroid
Noise Level	5.10^3
**ADAP Chromatogram builder**	
MS Level	1
Min. group size in # of scans	4
Group intensity threshold	5.10^4
Min highest intensity	5.10^4
m/z tolerance	0.002 *m/z* or 10 ppm
**Chromatogram resolving:** Local minimum resolver	
Chromatographic threshold	90%
Search minimum RT range	0.1 min
Minimum rel. Height	0
Minimum absol. Height	5.10^4
Ratio top/edge	1.9
Peak duration	0.0 - 10 min
Min # data points	3
**Join aligner**	
**Deisotoping**	
m/z tolerance	0.002 *m/z* or 10 ppm
Weight for m/z	75
Retention time tolerance	0.5 min
Weight for retention time	25
**Feature list filtering**	
Isotope tolerance	0.001/5ppm; 10^4, 80%
**Feature list blank filtering**	Min # detection 3
**Gap filling**	
Intensity tolerance	0.5 min
m/z tolerance	0.002 *m/z* or 10ppm
Retention time tolerance	0.5 min
**Peak list rows filter:** Feature with MS2 scan (GNPS)	yes
**Peak list rows filter:** Min features in row	2
**Duplicate feature filter**	0.002/5ppm; 2.0min
**Export for/Submit to GNPS**	

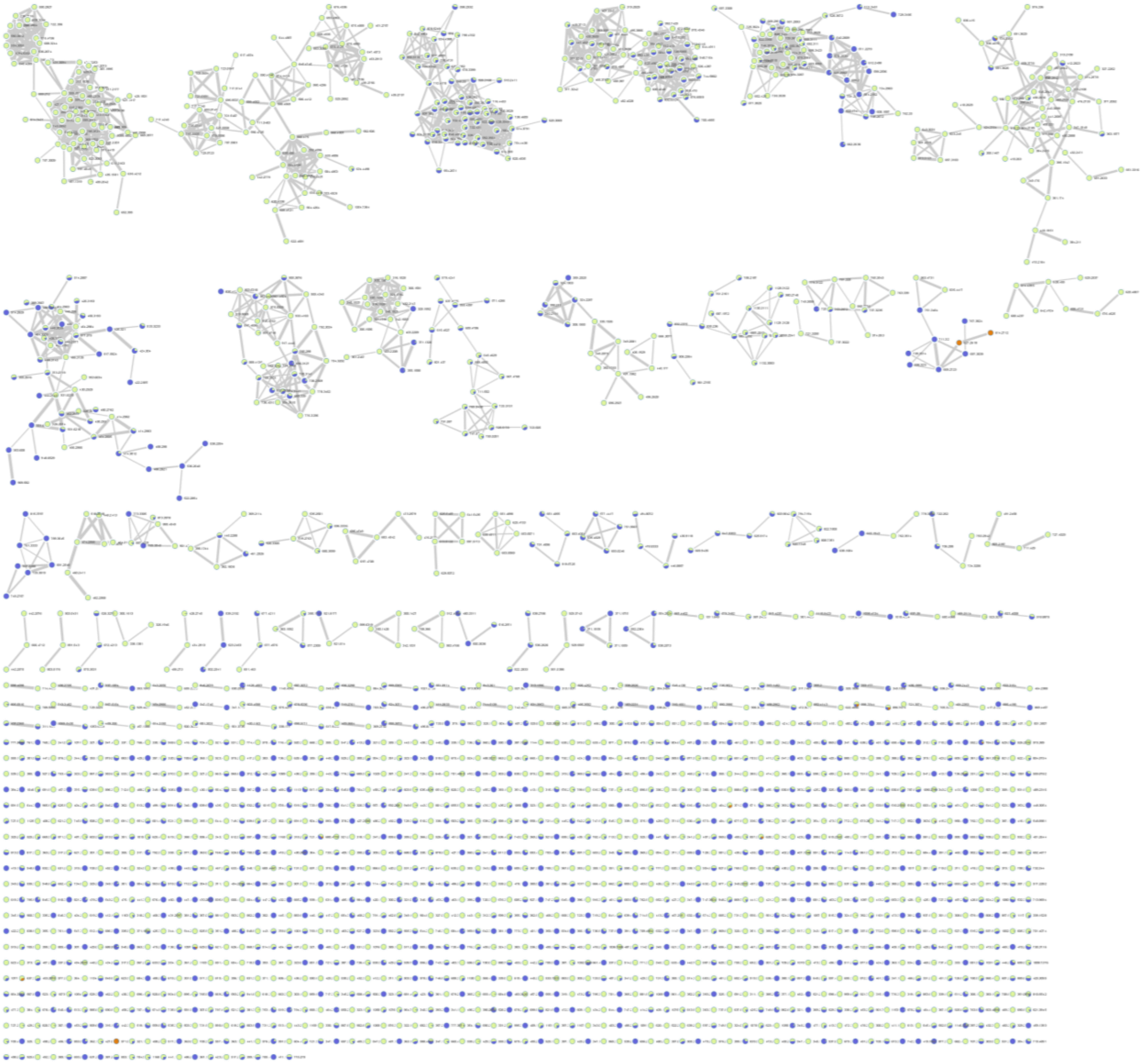

Figure S2: Global molecular network from Tenacibaculum maritimum LSP9.1. Nodes in green belong to the cell-pellet, while nodes in blue were extracted from the cell-free supernatant. In orange are represented the nodes corresponding the ferrioxamine D1, added as a standard.

Manual ion identity (MII) layer for data simplification

To improve interpretability and reduce redundancy in the molecular network, we manually applied an ion identity (MII) layer for all detected metal complexes.
First, we identified subnetworks containing metal-binding metabolites by screening for the characteristic Fe3+ and Ga3+ isotopic patterns. Candidate ions were curated in an Excel sheet along with their corresponding m/z values (Supporting Information 2). Using their MS1 spectra, we assigned the most probable adduct type of each ion (e.g. [M+H]+, [M+Na]+ or [M+K]+), and subsequently calculated the neutral monoisotopic mass. This neutral mass was used to calculate the molecular formula using in silico tools (e.g., SIRIUS or elemental composition calculators).
By comparing predicted formulae across adducts, we identified ions that represented the same underlying metabolite and manually grouped them under a shared ion identity. This enabled us to connect fragmented molecular families and simplify network topology, thereby facilitating more accurate structural annotation of metal-binding compounds.

Direct infusion ion mobility mass spectrometry and LOD determination

TMSX7M and TMPX7M fractions along with a solvent blank (9:1 H_2O:MeCN) were analyzed in a Trapped Ion Mobility Spectrometer Time of Flight (TIMS-TOF, Bruker) equipment with electrospray ionization. Immediately before the analysis, the exact mass at the TOF and the mobility at the TIMS were calibrated using the calibration solution ESI-L low concentration tuning Mix and the reference list of Tuning Mix ES-TOF CCS compendium. The infusion was performed with a 250 µL Hamilton syringe at a flow rate of 3.0 µL min^{-1} for analysis and calibration. The TOF module was calibrated using a quadratic calibration mode and positive ionization with the following list of reference masses: 322.0408 ($C_6H_{19}N_3O_6P_3^+$), 622.0290 ($C_{12}H_{19}F_{12}N_3O_6P_3^+$), 922.0098 ($C_{18}H_{19}F_{24}N_3O_6P_3^+$), and 1221.9906 ($C_{24}H_{19}F_{36}N_3O_6P_3^+$). Parallelly, TIMS was calibrated following a linear calibration mode until a score of 100% was reached using the corrected values of $1/K_0$: 0.985 (for $C_{12}H_{19}F_{12}N_3O_6P_3^+$), 1.190 (for $C_{18}H_{19}F_{24}N_3O_6P_3^+$), and 1.382 V.s.$cm^{-2}$ (for $C_{24}H_{19}F_{36}N_3O_6P_3^+$).

Analysis of the samples and blanks was performed in the ranges of mass *m/z* 300-1300, and mobility of $1/K_0$ 0.6 – 1.56 V s cm^{-2}. An absolute threshold of 38 counts per 100 ms Accu time and an absolute intensity threshold of 10^5 counts were fixed for the mass spectra peak detection and mobilogram peak detection, respectively. The ESI source was set with an end plate offset of 500 V and an electrospray voltage of +4000 V for positive polarization. Nebulizer, dry gas flow, and dry gas temperature were established at 0.4 bar, 4 L min^{-1}, and 250 ºC, respectively. The transfer was tuned with the following parameters: deflection 1 delta of 80 V, funnel 1RF and 2RF of 250.0 Vpp, isCID energy of 0 eV, and multipole RF of 200 Vpp. Similarly, the tune of the quadrupole was set with an ion energy of 5 eV and a low mass of *m/z* 300, while the collision cell was fixed to a collision energy of 10 eV, a collision RF of 800 Vpp, a transfer time of 65 µs and pre-pulse storage of 5 µs. For the TIMS analyzer, the ion charge control (ICC) was activated and set to 2.00 mio to prevent saturation, the ramp time was set to 260.8 ms, and the spectra rate to 3.75 Hz. TIMS was tuned with a funnel 1 RF of 250 Vpp and a collision cell of 220 V. The different delta values of the TIMS tuning were the following: Δt1 of -20 V, Δt2 of -120 V, Δt3 of 80 V, Δt4 of 100 V, Δt5 of 0 V, and Δt6 of 100 V. Three independent replicates of TMSX7M and TMPX7M fractions were analyzed.

The limit of detection (LOD) was determined using a calibration curve constructed by plotting the intensity of ferrioxamine B (*m/z* 614.2720) over a concentration range of 9×10^{-3} to 9×10^{-9} mg/mL. The LOD value of 7.9 pg/mL was calculated using the following equation:

$$LOD = 3.3(Sy/S)$$

where *Sy* is the standard deviation of the blank responses and *S* is the slope of the calibration curve.

2. Direct-injection ion mobility spectrometry validation

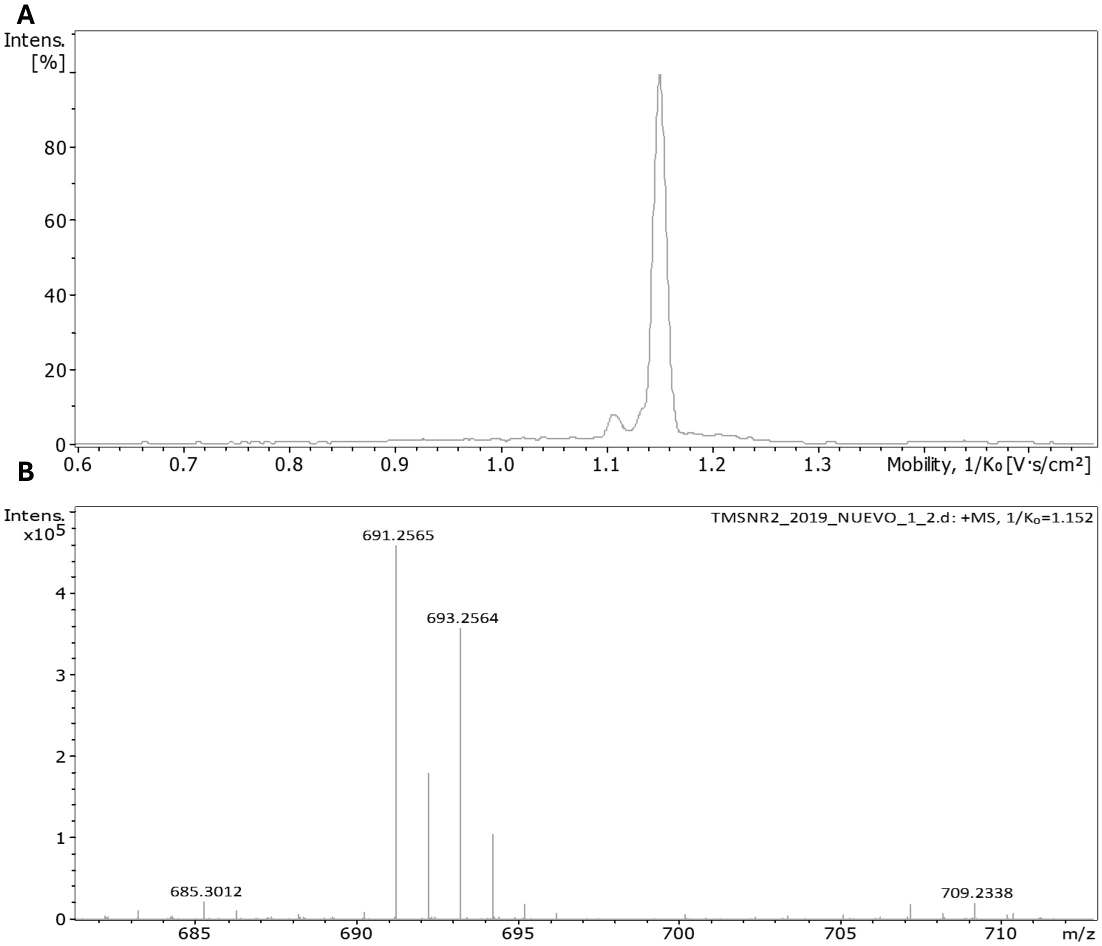

Figure S3: DI-IMS-MS validation of desferrioxamine D1 (**2**): (**A**) Ion mobilogram showing a mobility of $1/K_0$ [V·s/cm^2] = 1.152. (**B**) (+)-HRMS spectrum of the $[M\text{-}3H\text{+}Ga\text{+}Na]^+$ ion at *m/z* 691.2565.

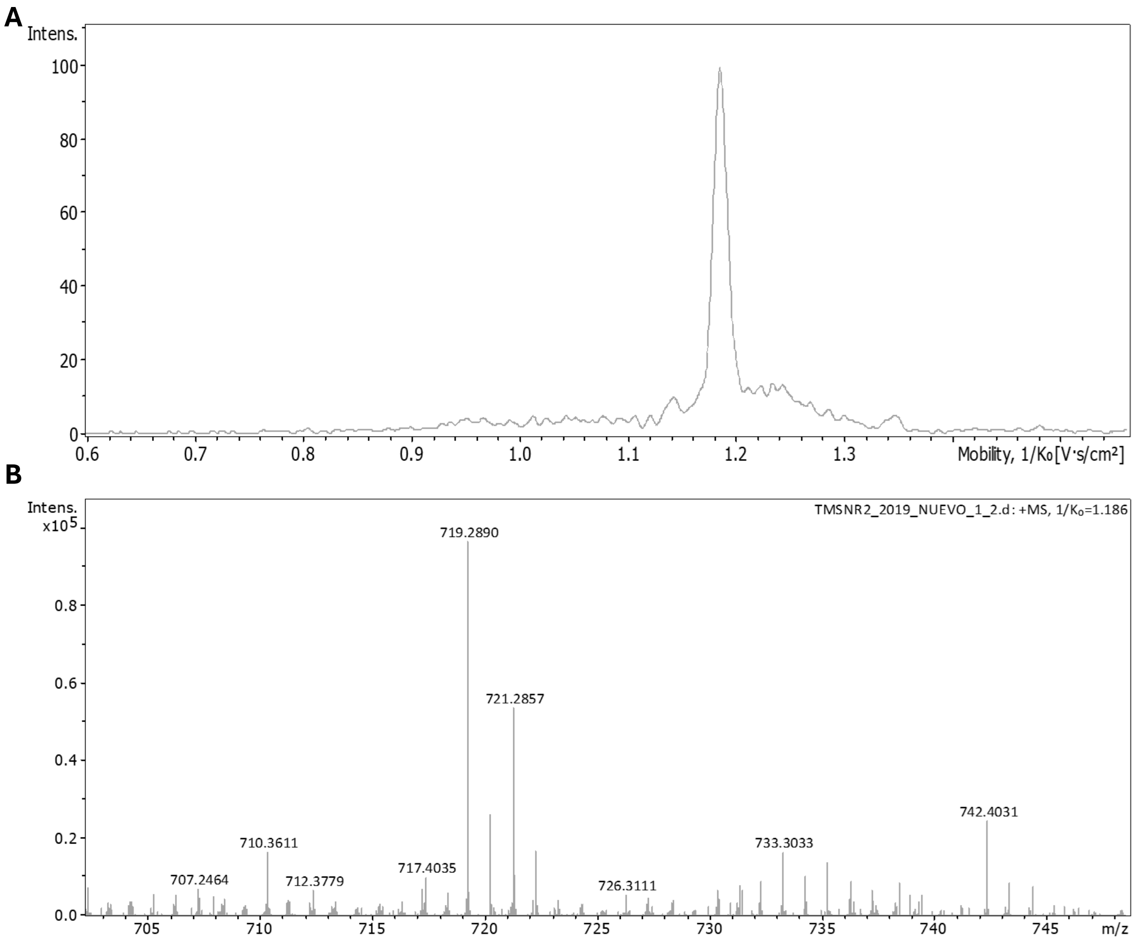

Figure S4: DI-IMS-MS validation of C4 acyl DFO-D (**3**): (**A**) Ion mobilogram showing a mobility of $1/K_0$ [$V{\cdot}s/cm^2$] = 1.186. (**B**) (+)-HRMS spectrum of the [M-3H+Ga+Na]* ion at *m/z* 719.2890.

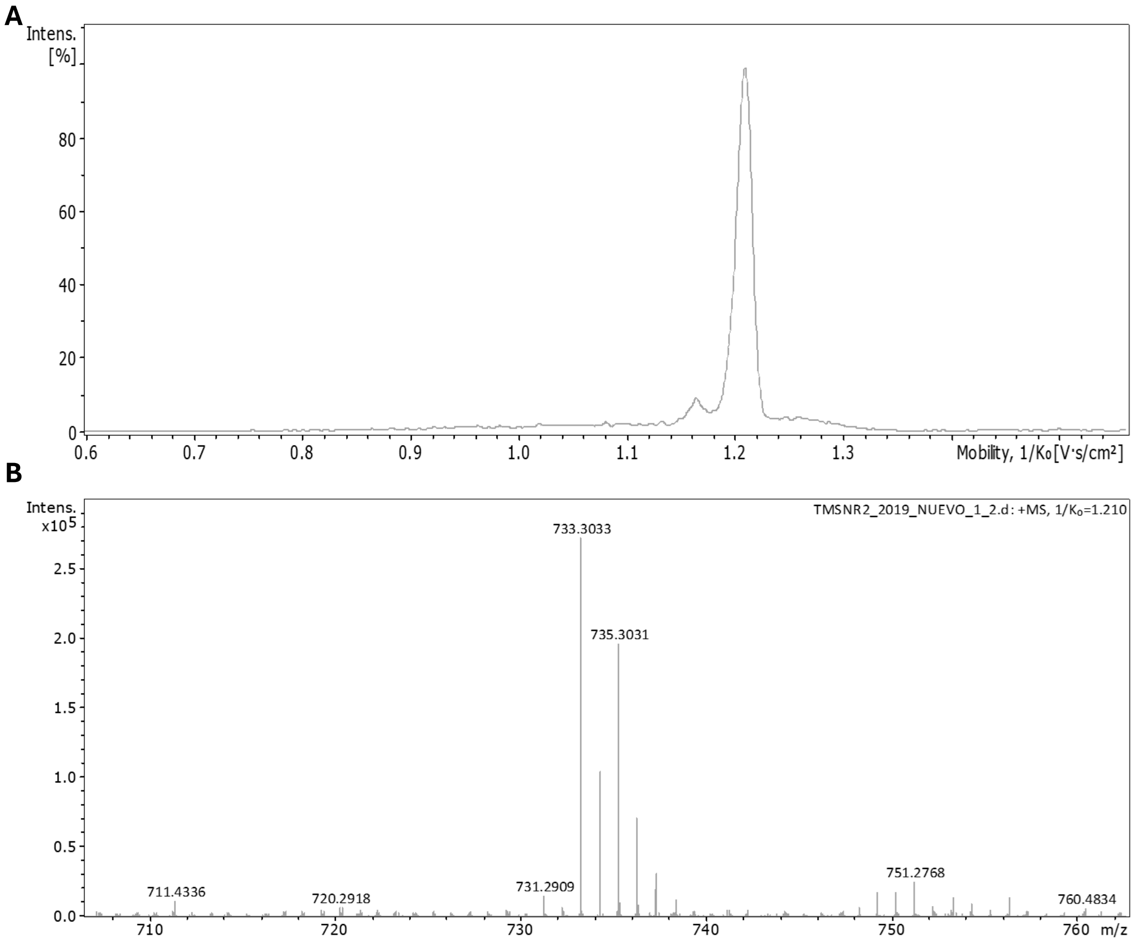

Figure S5: DI-IMS-MS validation of C5 acyl DFO-D (**4**): (**A**) Ion mobilogram showing a mobility of $1/K_0$ [V·s/cm^2] = 1.210. (**B**) (+)-HRMS spectrum of the $[M\text{-}3H\text{+}Ga\text{+}Na]^+$ ion at *m/z* 733.3033.

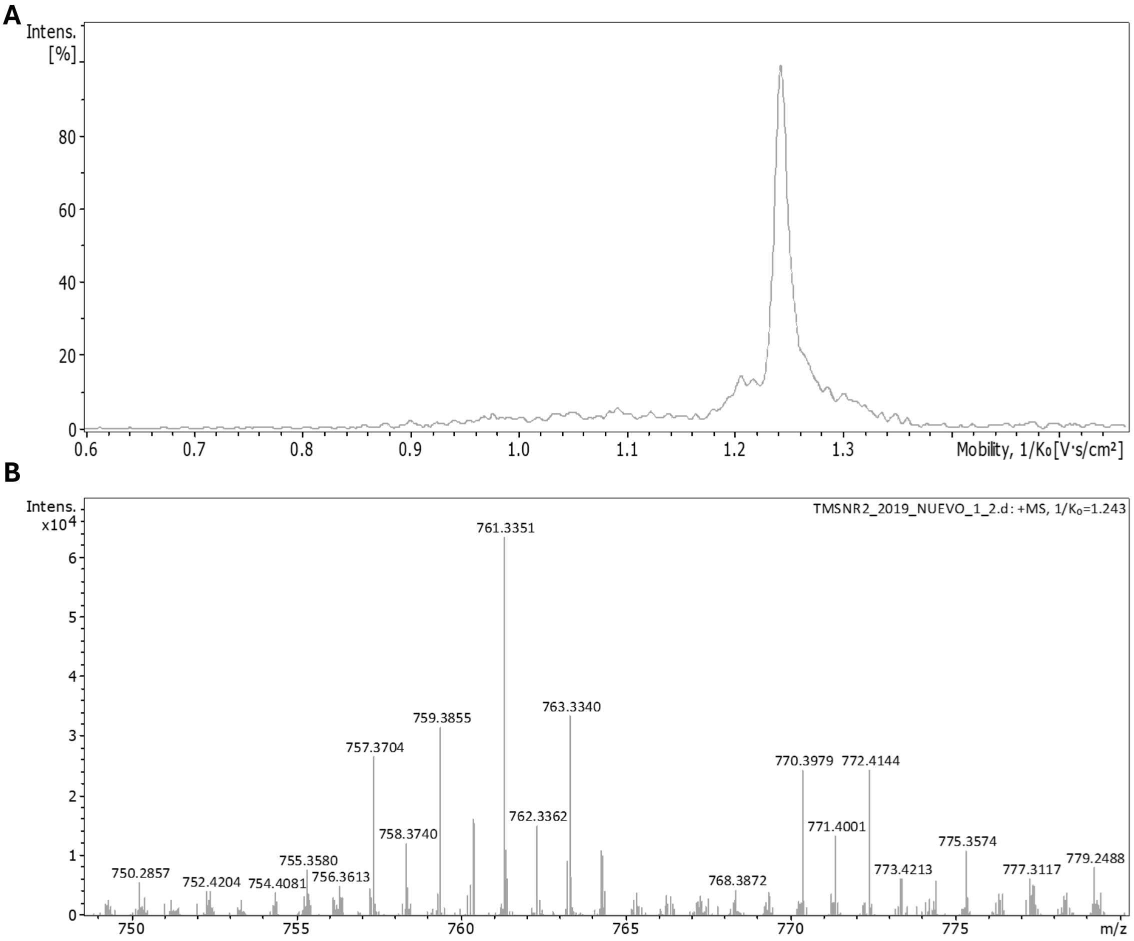

Figure S6: DI-IMS-MS validation of C7 acyl DFO-D (**5**): (**A**) Ion mobilogram showing a mobility of $1/K_0$ [V·s/cm^2] = 1.243. (**B**) (+)-HRMS spectrum of the $[M\text{-}3H+Ga+Na]^+$ ion at *m/z* 761.3351.

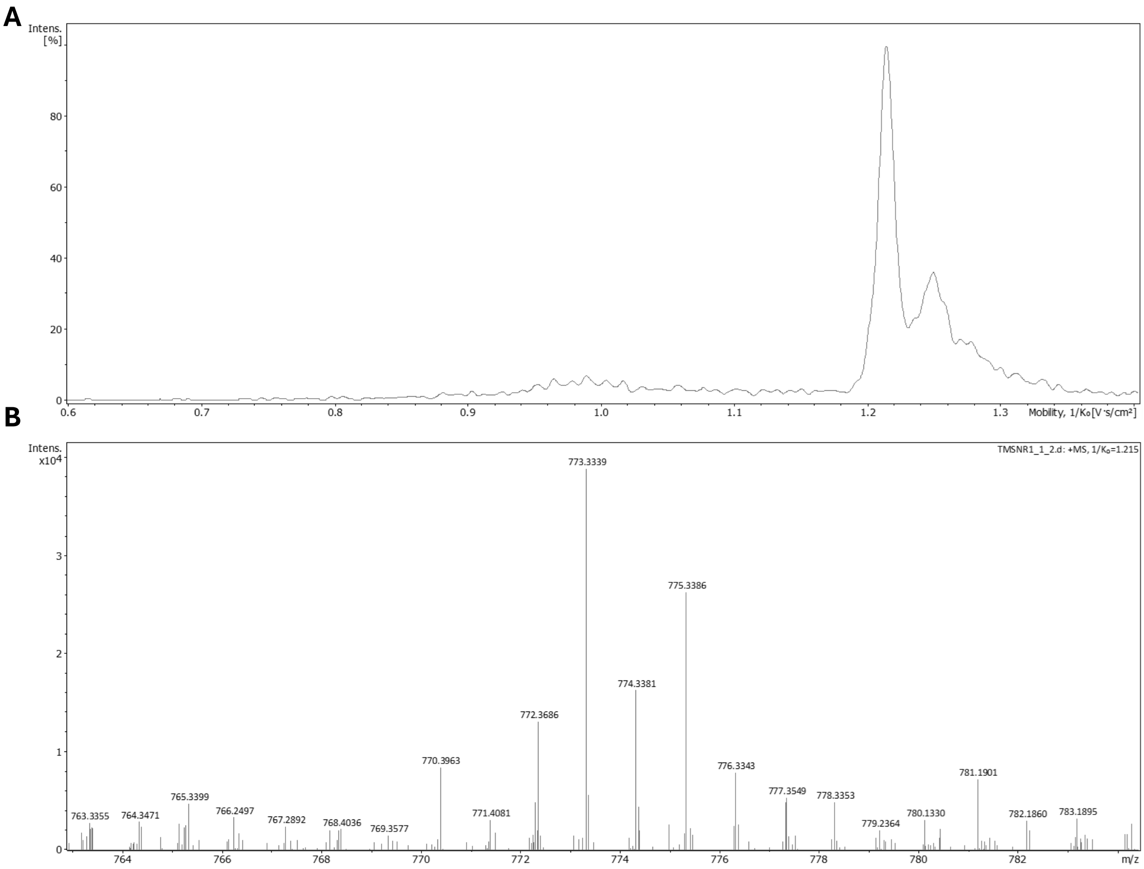

Figure S7: DI-IMS-MS validation of C8:1 acyl DFO-D (**6**): (**A**) Ion mobilogram showing a mobility of $1/K_0$ [V·s/cm^2] = 1.215 (**B**) (+)-HRMS spectrum of the [M-3H+Ga+Na]$^+$ ion at *m/z* 773.3339.

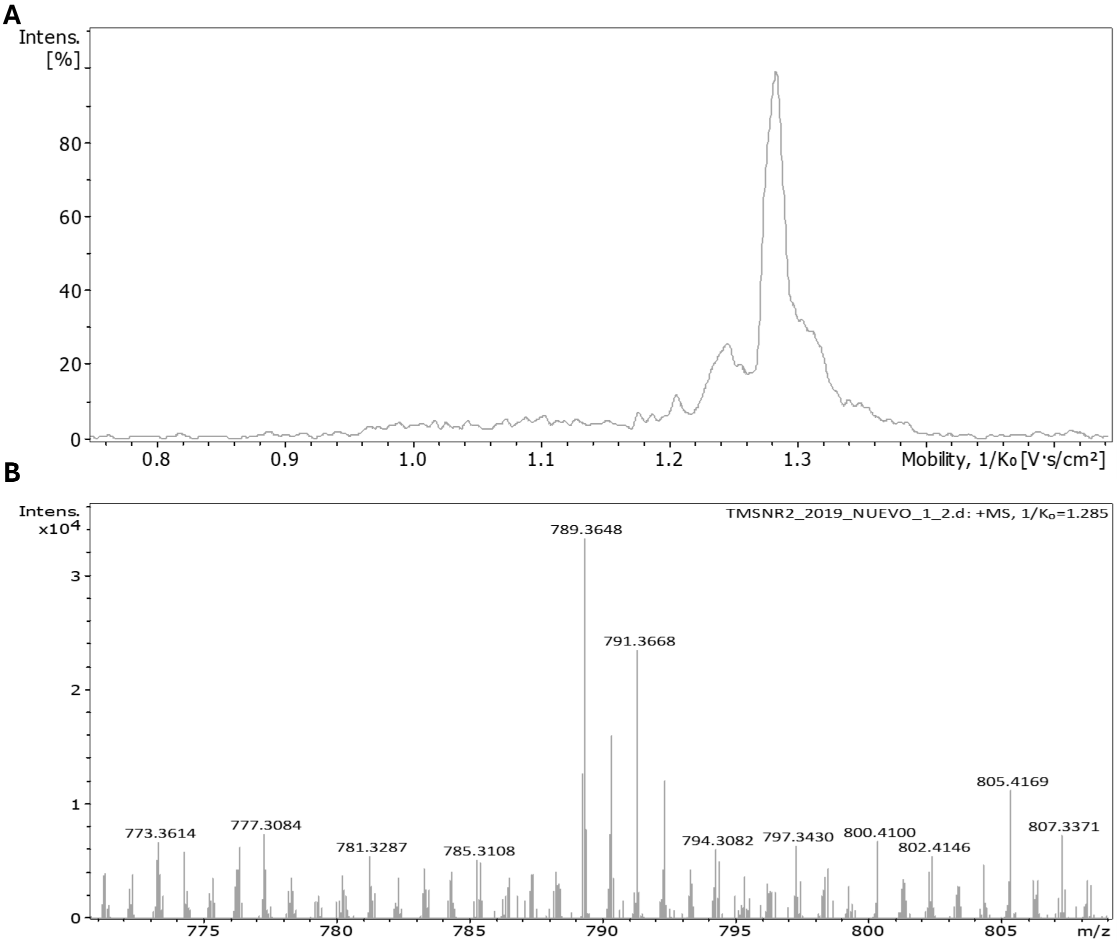

Figure S7: DI-IMS-MS validation of C9 acyl DFO-D (**7**): (**A**) Ion mobilogram showing a mobility of $1/K_0$ [V·s/cm^2] = 1.285. (**B**) (+)-HRMS spectrum of the [M-3H+Ga+Na]$^+$ ion at *m/z* 789.3648.

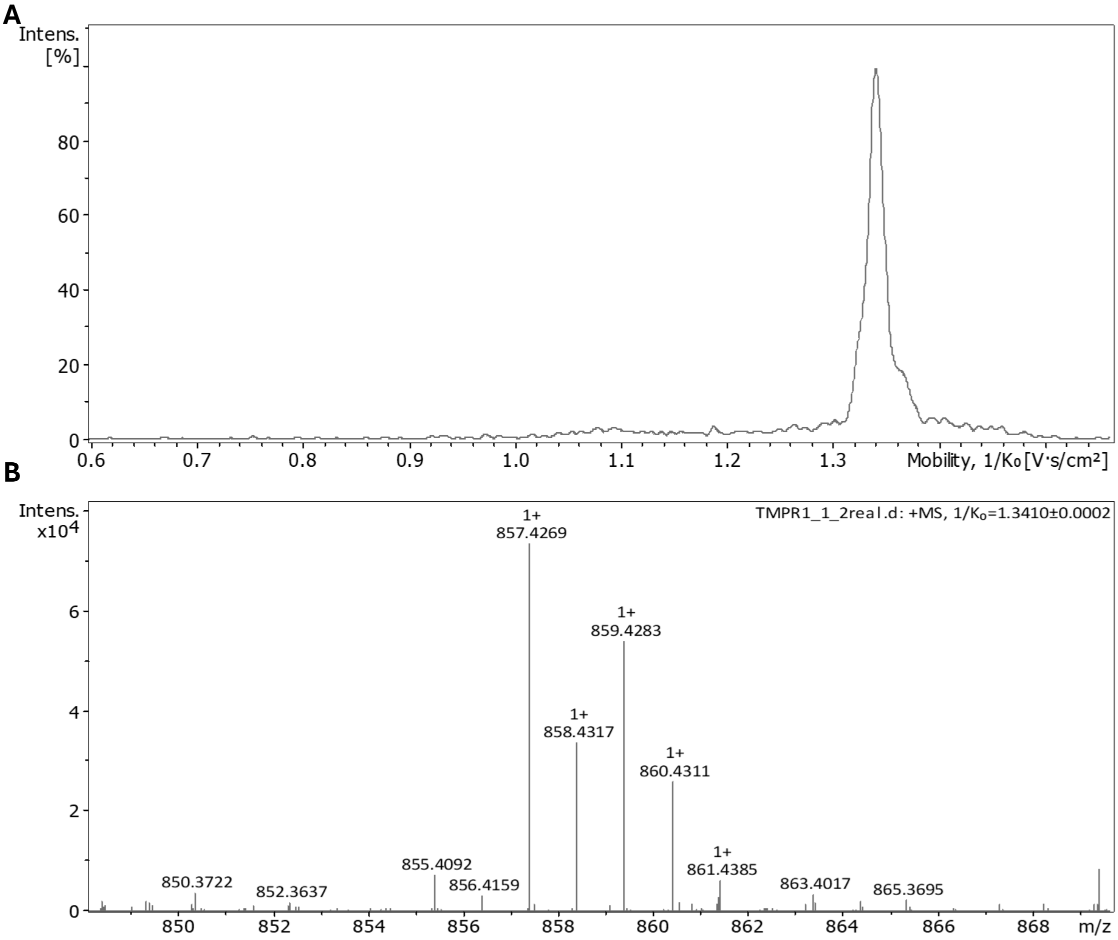

Figure S8: DI-IMS-MS validation of C14:1 acyl DFO-D (**8**): (**A**) Ion mobilogram showing a mobility of $1/K_0$ [V·s/cm^2] = 1.341. (**B**) (+)-HRMS spectrum of the [M-3H+Ga+Na]$^+$ ion at *m/z* 857.4269.

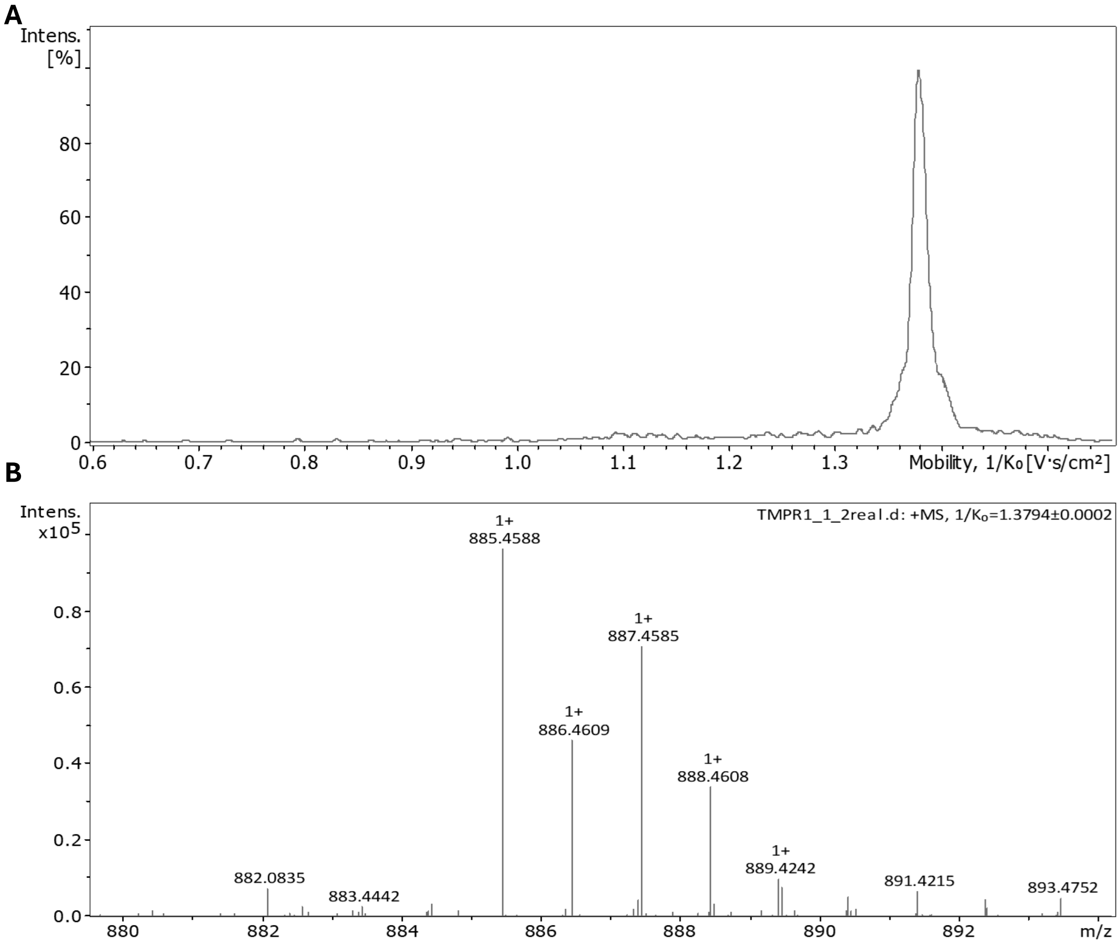

Figure S9: DI-IMS-MS validation of C16:1 acyl DFO-D (**9**): (**A**) Ion mobilogram showing a mobility of $1/K_0$ [V·s/cm^2] = 1.379. (**B**) (+)-HRMS spectrum of the $[M-3H+Ga+Na]^+$ ion at *m/z* 885.4588.

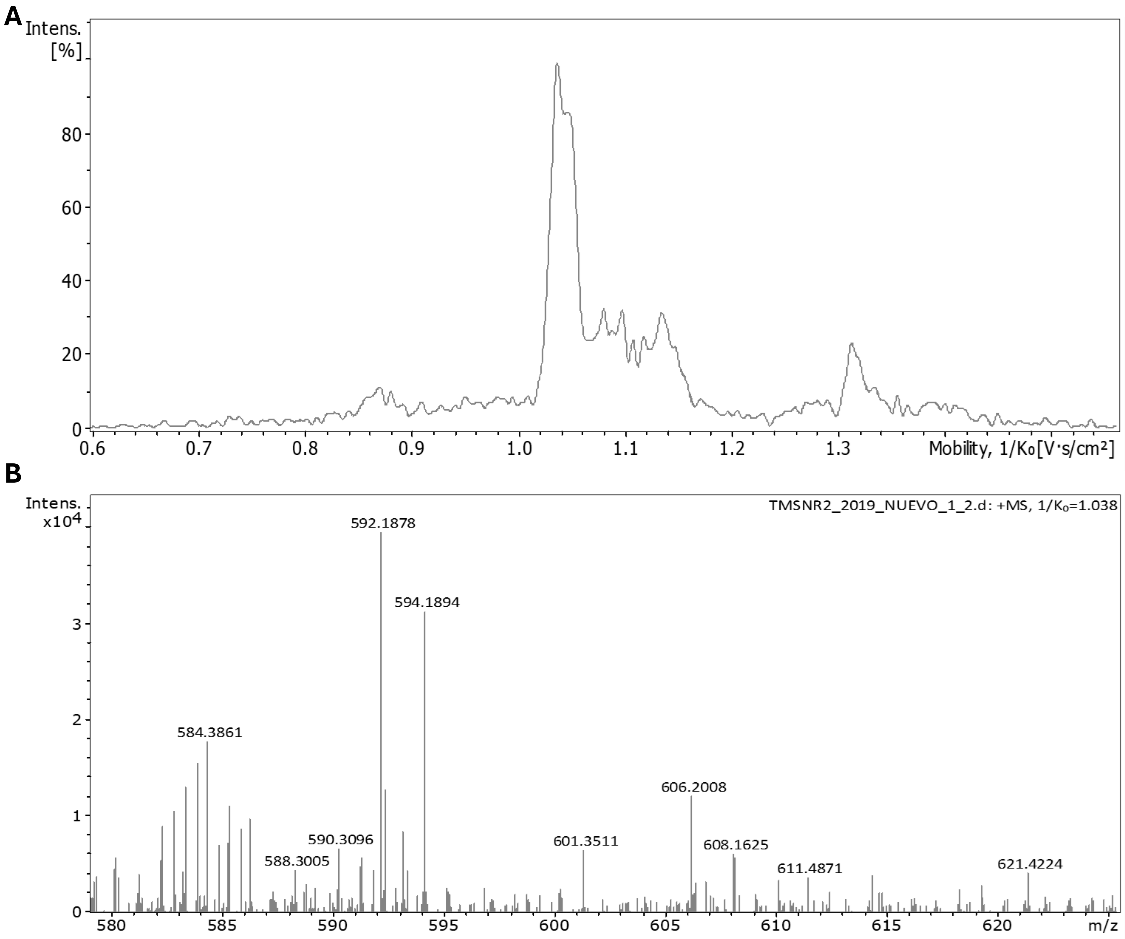

Figure S10: DI-IMS-MS validation of compound **10**: (**A**) Ion mobilogram showing a mobility of $1/K_0$ [V·s/cm^2] = 1.152. (**B**) (+)-HRMS spectrum of the [M-3H+Ga+Na]$^+$ ion at *m/z* 592.1878.

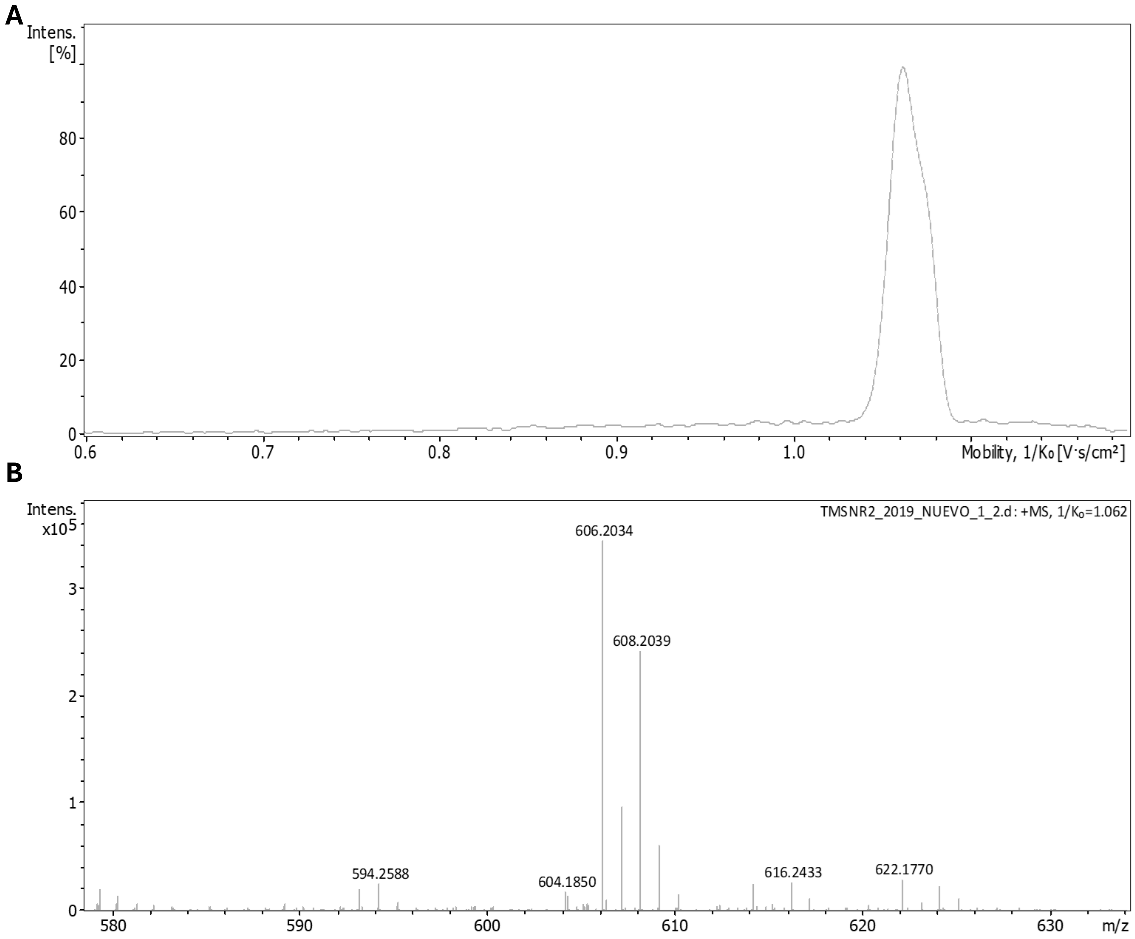

Figure S11: DI-IMS-MS validation of compound **11**: (**A**) Ion mobilogram showing a mobility of $1/K_0$ [V·s/cm^2] = 1.062. (**B**) (+)-HRMS spectrum of the [M-3H+Ga+Na]$^+$ ion at *m/z* 606.2034.

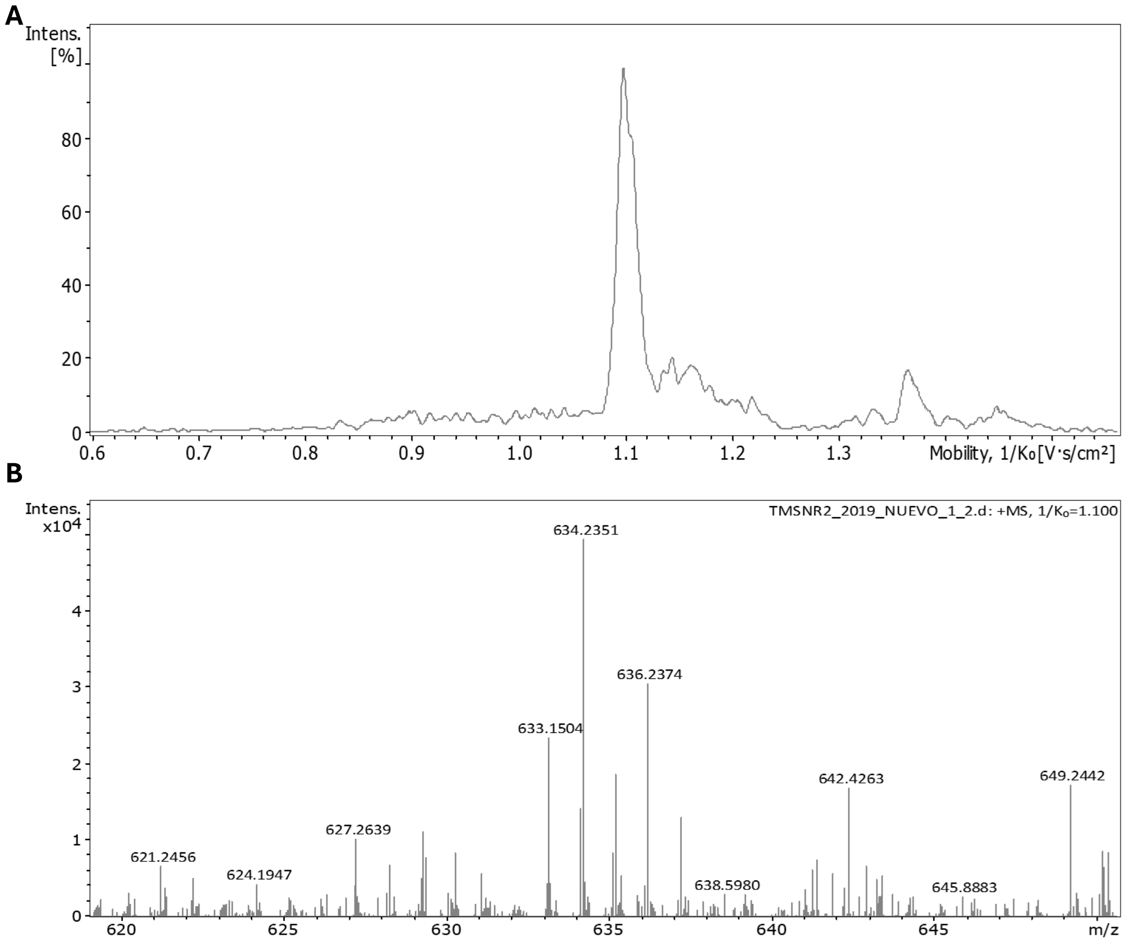

Figure S12: DI-IMS-MS validation of compound **12**: (**A**) Ion mobilogram showing a mobility of $1/K_0$ [V·s/cm^2] = 1.100. (**B**) (+)-HRMS spectrum of the [M-3H+Ga+Na]$^+$ ion at *m/z* 634.2351.

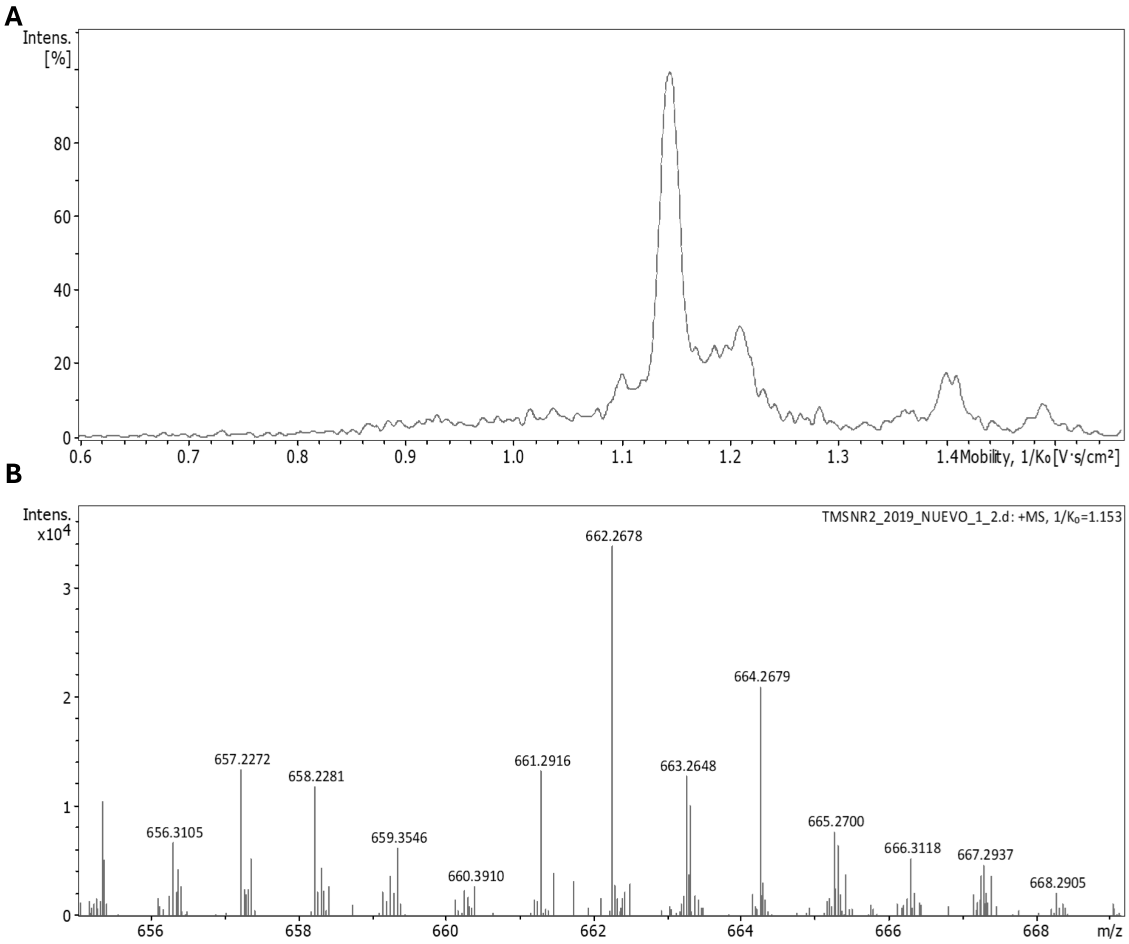

Figure S13: DI-IMS-MS validation of compound **13**: (**A**) Ion mobilogram showing a mobility of $1/K_0$ [V·s/cm^2] = 1.153. (**B**) (+)-HRMS spectrum of the [M-3H+Ga+Na]$^+$ ion at *m/z* 662.2678.

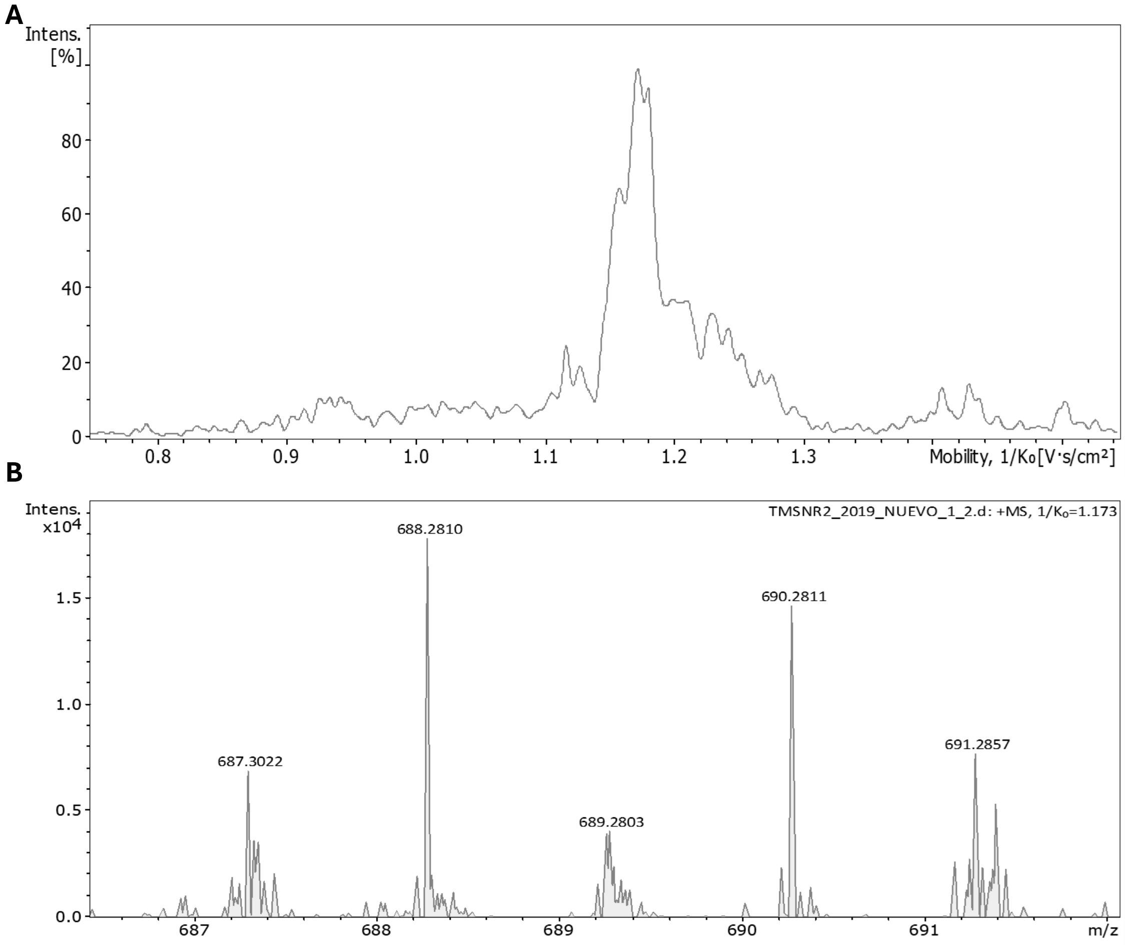

Figure S14: DI-IMS-MS validation of compound **14**: (**A**) Ion mobilogram showing a mobility of $1/K_0$ [V·s/cm^2] = 1.173. (**B**) (+)-HRMS spectrum of the [M-3H+Ga+Na]$^+$ ion at *m/z* 688.2817.

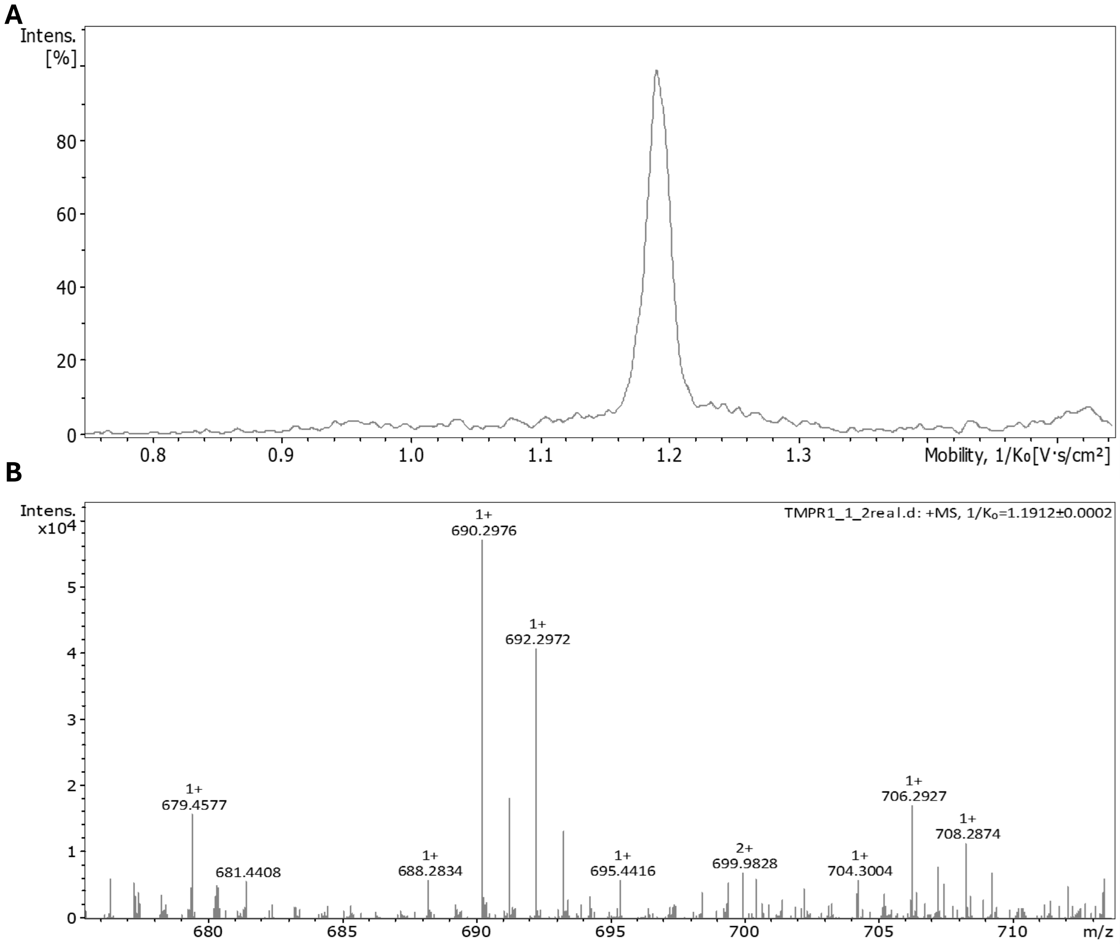

Figure S15: DI-IMS-MS validation of compound **15**: (**A**) Ion mobilogram showing a mobility of $1/K_0$ [V·s/cm^2] = 1.191. (**B**) (+)-HRMS spectrum of the [M-3H+Ga+Na]$^+$ ion at *m/z* 690.2976.

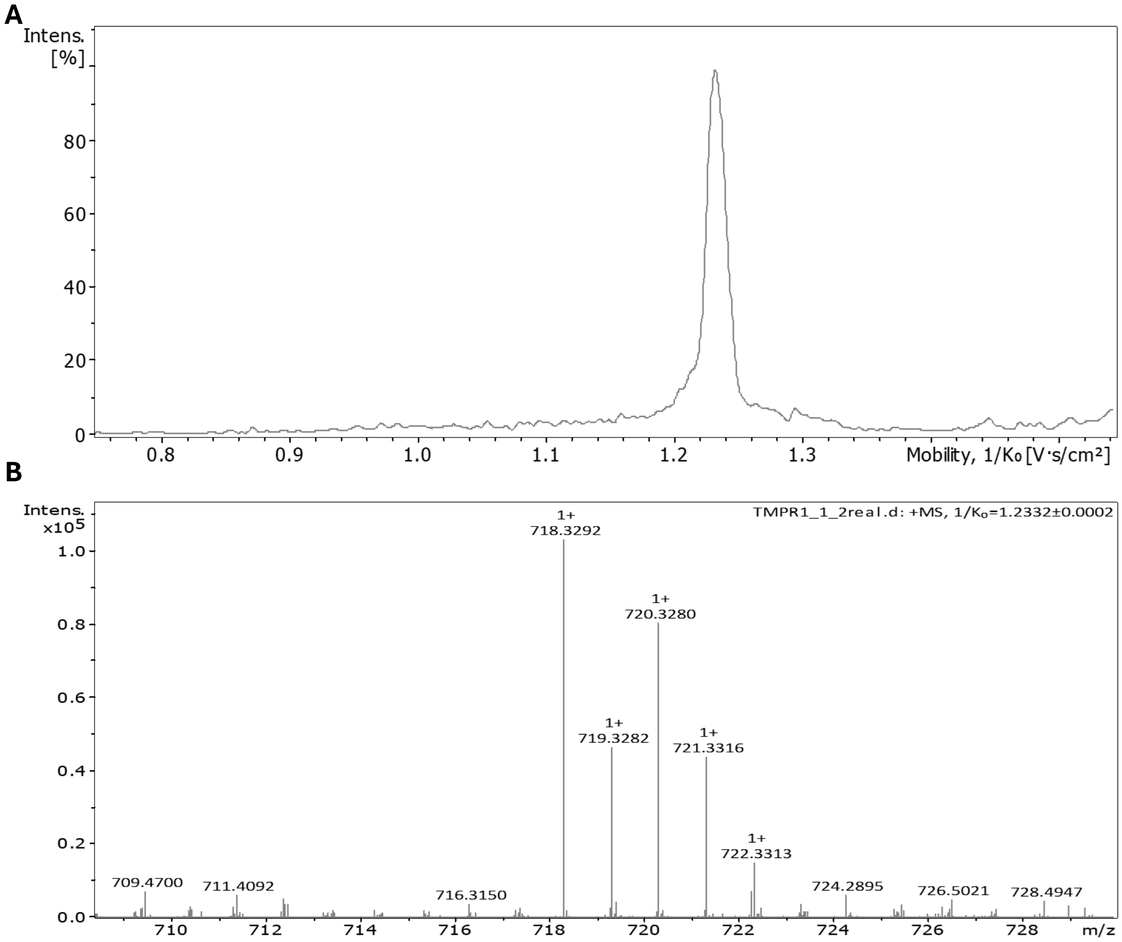

Figure S16: DI-IMS-MS validation of compound **16**: (**A**) Ion mobilogram showing a mobility of $1/K_0$ [V·s/cm^2] = 1.233. (**B**) (+)-HRMS spectrum of the [M-3H+Ga+Na]$^+$ ion at *m/z* 718.3292.

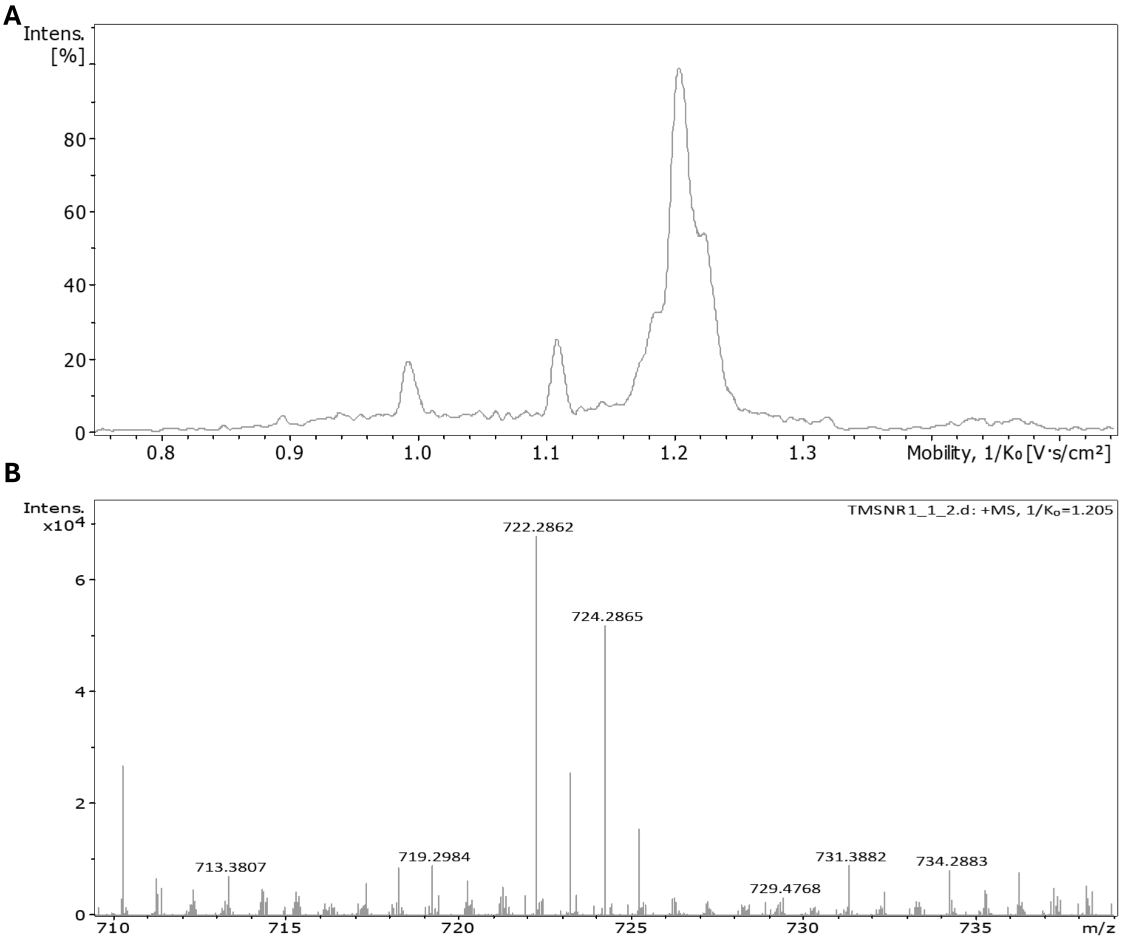

Figure S17: DI-IMS-MS validation of compound **17**: (**A**) Ion mobilogram showing a mobility of $1/K_0$ [V·s/cm^2] = 1.205. (**B**) (+)-HRMS spectrum of the [M-3H+Ga+Na]$^+$ ion at *m/z* 722.2862.

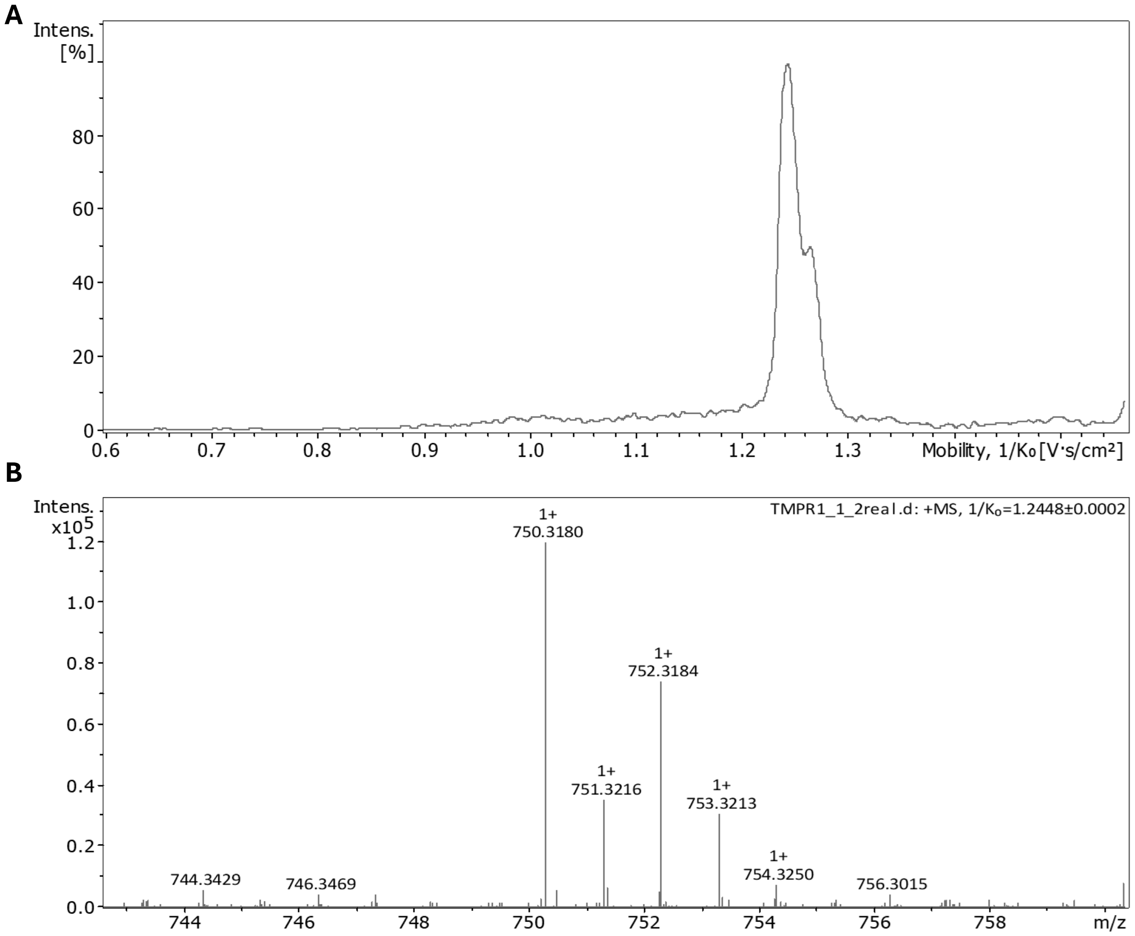

Figure S18: DI-IMS-MS validation of compound **18**: (**A**) Ion mobilogram showing a mobility of $1/K_0$ [V·s/cm^2] = 1.245. (**B**) (+)-HRMS spectrum of the [M-3H+Ga+Na]$^+$ ion at *m/z* 750.3180.

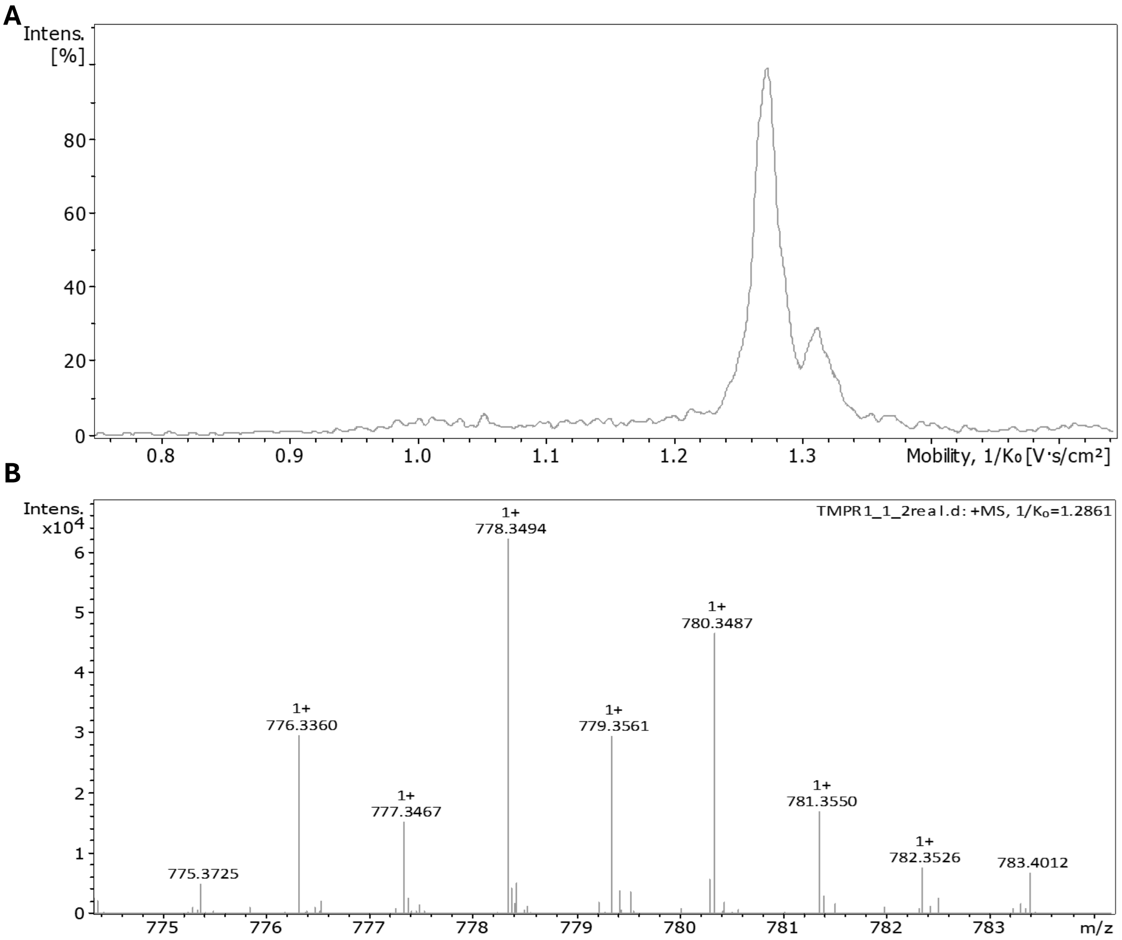

Figure S19: DI-IMS-MS validation of compound **19**: (**A**) Ion mobilogram showing a mobility of $1/K_0$ [V·s/cm^2] = 1.2861. (**B**) (+)-HRMS spectrum of the $[M-3H+Ga+Na]^+$ ion at *m/z* 778.3494.

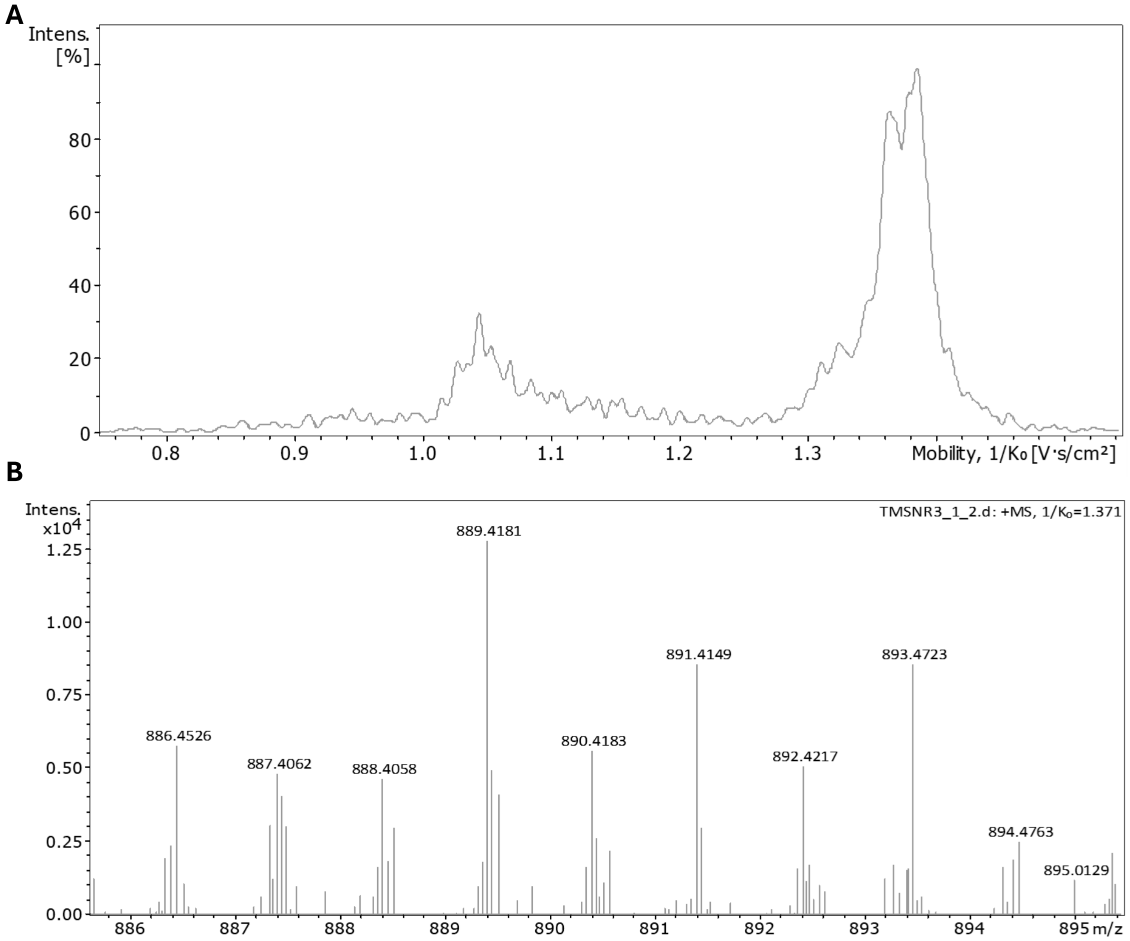

Figure S20: DI-IMS-MS validation of compound **20**: (**A**) Ion mobilogram showing a mobility of $1/K_0$ [V·s/cm^2] = 1.371. (**B**) (+)-HRMS spectrum of the [M-3H+Ga+Na]$^+$ ion at *m/z* 889.4182.

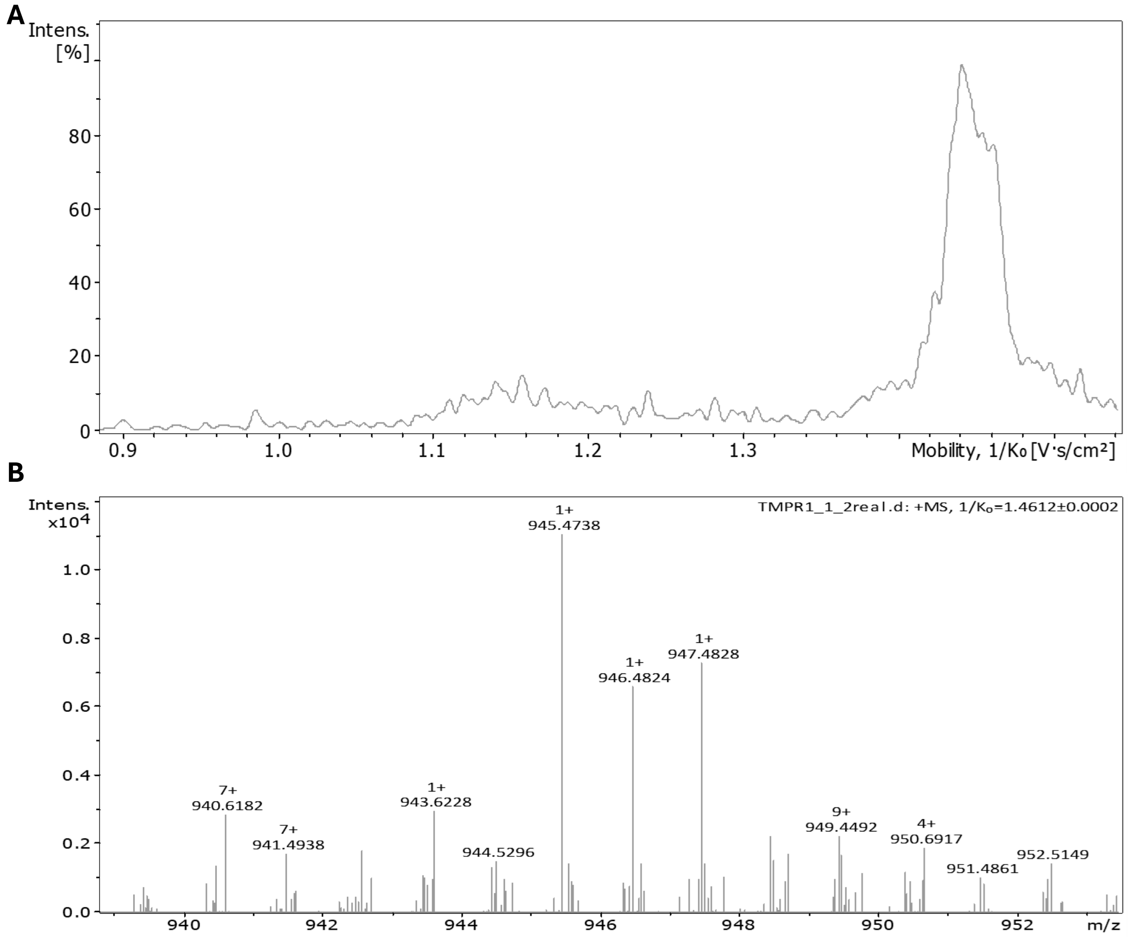

Figure S21: DI-IMS-MS validation of compound **21**: (**A**) Ion mobilogram showing a mobility of $1/K_0$ [V·s/cm^2] = 1.152. (**B**) (+)-HRMS spectrum of the [M-3H+Ga+Na]$^+$ ion at *m/z* 917.4507.

3. MS/MS fragmentation patterns

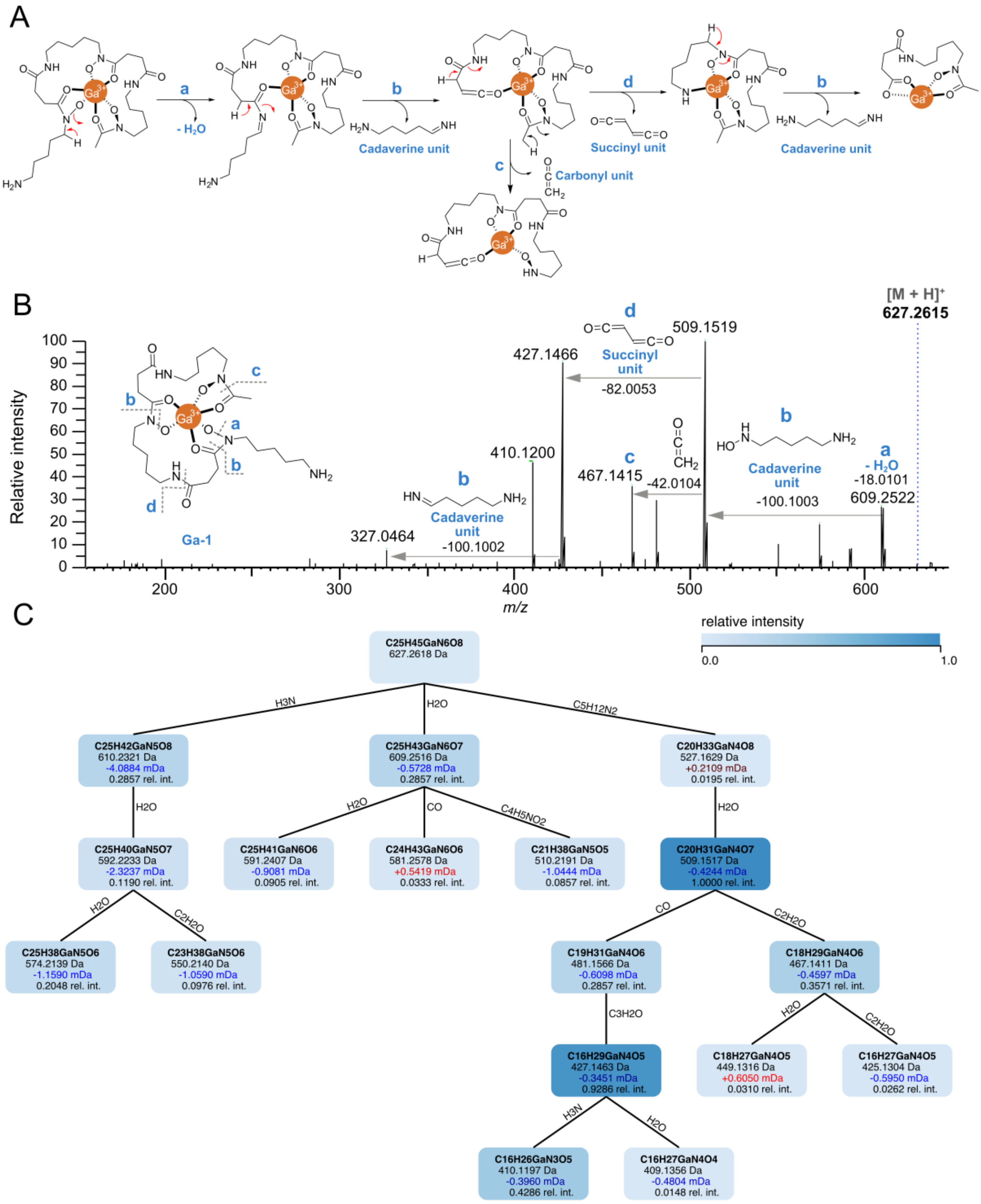

Figure S22: MS/MS fragmentation pattern of desferrioxamine B (**1**) chelated with gallium (**Ga-1**): (**A**) Proposed fragmentation pathway of **Ga-1** based on remote hydrogen rearrangements, (**B**) MS/MS spectrum from the [M + H]+ ion of **Ga-1**, and (**C**) proposed fragmentation tree by SIRIUS 5.8.3.

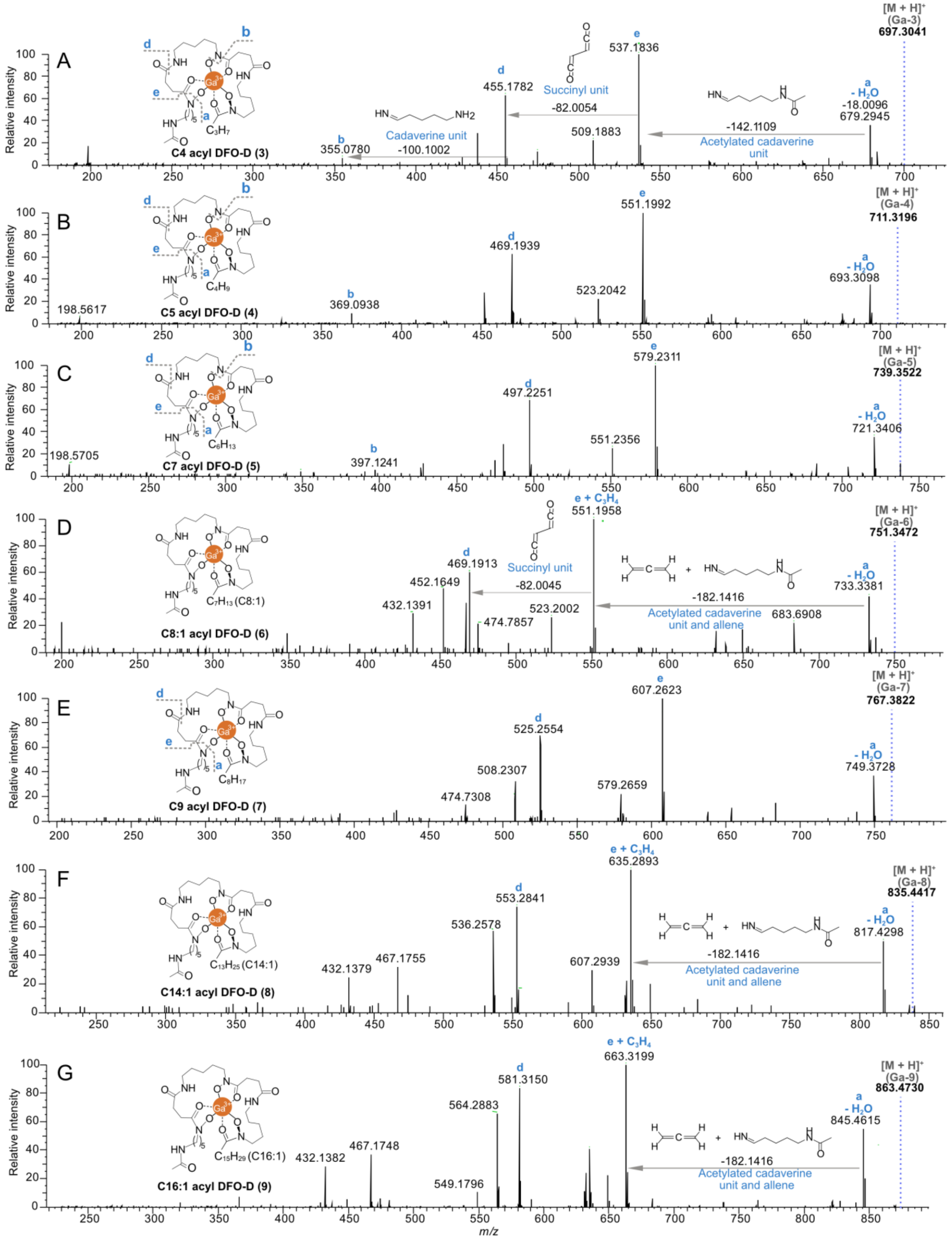

Figure S23: MS/MS fragmentation pattern from the $[M - 2H + Ga]^{+}$ ions of the metallophores **A**) C4 acyl DFO-D (**3**), **B**) C5 acyl DFO-D (**4**), **C**) C7 acyl DFO-D (**5**), **D**) C8:1 acyl DFO-D (**6**), **E**) C9 acyl DFO-D (7), **F**) C14:1acyl DFO-D (**6**), and **G**) C16:1 acyl DFO-D (**9**).

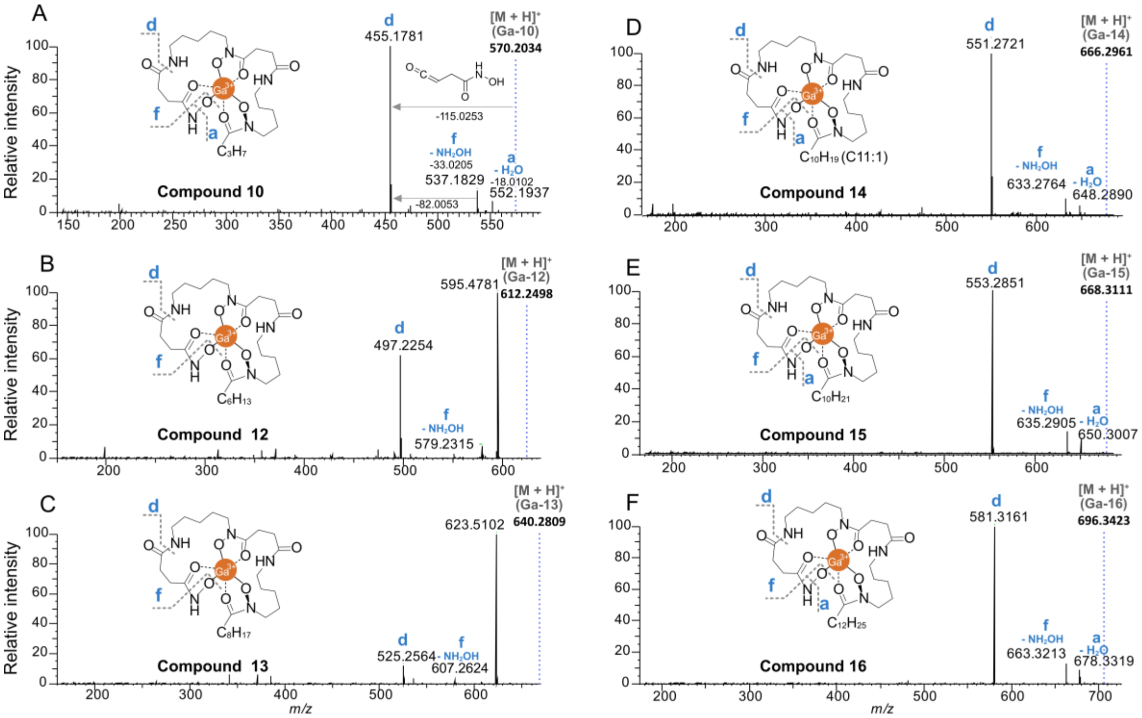

Figure S24: MS/MS fragmentation pattern from the [M - 2H + Ga]$^+$ ions of the metallophores: **A**) compound **10**, **B**) compound **12**, **C**) compound **13**, **D**) compound **14**, **E**) compound **15**, and **F**) compound **16**.

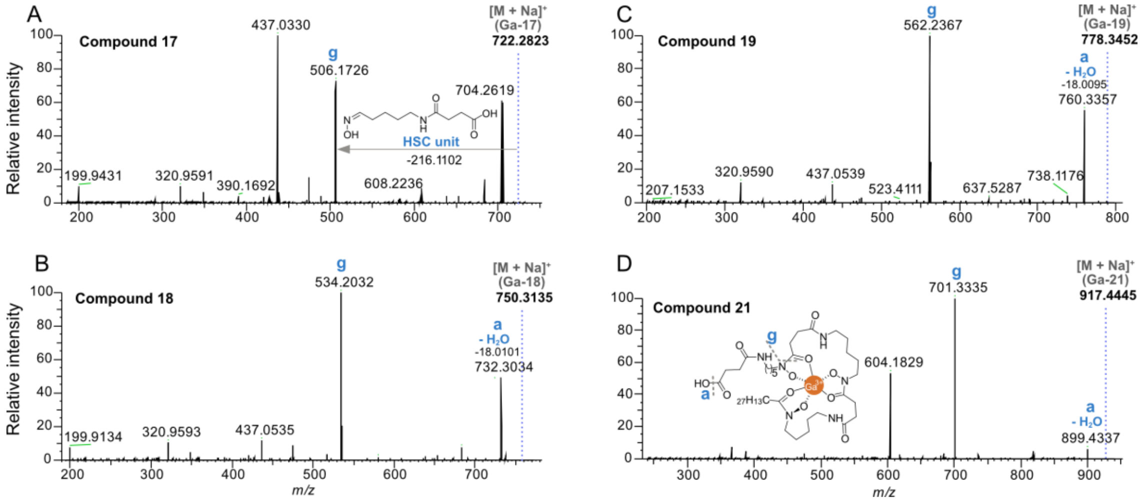

Figure S25: MS/MS fragmentation pattern from the [M - 3H + Ga + Na]$^+$ ions of the metallophores: **A**) compound **17**, **B**) compound **18**, **C**) compound **19**, and **D**) compound **21**.

Table S3: Chemical formulae, feature-based molecular networking, and DI-IMS-MS data of the metallophores found in the pellet and supernatant of *Tenacibaculum maritimum* LSP9.1

Metallophore	Chemical formula	Calculated monisotopic mass	Feature-based molecular networking					DI-IMS-MS validation		
			Experimental monoisotopic mass	Δ ppm	Subnetwork	Pellet	Supernatant	$[M - 3H + Ga + Na]^+$	Experimental monoisotopic mass	Δ ppm
				***N*-acetylated terminal siderophores**						
**Desferrioxamine D1 (2)**	$C_{27}H_{50}N_6O_9$	602.3639	602.363	-1.49	S1 and S2		X	691.2565	602.3647	1.28
**C4 acyl DFO-D (3)**	$C_{29}H_{54}N_6O_9$	630.3952	630.3939	-2.06	S1		X	719.289	630.3972	3.13
**C5 acyl DFO-D (4)**	$C_{30}H_{56}N_6O_9$	644.4109	644.4101	-1.24	S1 and S2		X	733.3033	644.4115	0.89
**C7 acyl DFO-D (5)**	$C_{32}H_{60}N_6O_9$	672.4422	672.4414	-1.19	S1		X	761.3351	672.4433	1.59
**C8:1 acyl DFO-D (6)**	$C_{33}H_{57}N_6O_9$	684.4421	684.4386	-5.11	S1		X	773.3351	684.4433	1.71
**C9 acyl DFO-D (7)**	$C_{34}H_{64}N_6O_9$	700.4735	700.4727	-1.18	S1		X	789.3648	700.4730	-0.76
**C14:1 acyl DFO-D (8)**	$C_{39}H_{72}N_6O_9$	768.5361	768.5318	-5.60	S1	X	X	857.4269	768.5351	-1.34
**C16:1 acyl DFO-D (9)**	$C_{41}H_{76}N_6O_9$	796.5674	796.5632	-5.27	S1	X		885.4588	796.5670	-0.54
				**Hydroxamic acid terminal siderophores**						
**Compound 10**	$C_{22}H_{41}N_5O_8$	503.2955	503.2937	-3.58	S4	X	X	592.1878	503.2960	0.94
**Compound 11**	$C_{23}H_{43}N_5O_8$	517.3112	517.308	-6.19	S4	X	X	606.2034	517.3116	0.72
**Compound 12**	$C_{25}H_{47}N_5O_8$	545.3425	545.3400	-4.58	S4	X	X	634.2351	545.3433	1.41
**Compound 13**	$C_{27}H_{51}N_5O_8$	573.3738	573.3713	-4.36	S4	X	X	662.2678	573.3760	3.79
**Compound 14**	$C_{29}H_{53}N_5O_8$	599.3894	599.3863	-5.17	S4	X	X	688.2817	599.3899	0.79
**Compound 15**	$C_{29}H_{55}N_5O_8$	601.4051	601.4015	-5.99	S4	X	X	690.2976	601.4058	1.12
**Compound 16**	$C_{31}H_{59}N_5O_8$	629.4364	629.4339	-3.97	S4	X		718.3292	629.4374	1.54
				***N*-succinic acid terminal siderophores**						
**Compound 17**	$C_{29}H_{55}N_5O_{10}$	633.3949	633.3912	-5.84	S6 and S7	X	X	722.2862	633.3944	-0.84
**Compound 18**	$C_{31}H_{59}N_5O_{10}$	661.4262	661.4226	-5.49	S6 and S7	X	X	750.318	661.4262	-0.04
**Compound 19**	$C_{33}H_{63}N_5O_{10}$	689.4575	689.4545	-4.35	S6 and S7	X	X	778.3494	689.4576	0.10
**Compound 20**	$C_{39}H_{72}N_6O_{11}$	800.5259	800.5218	-5.08	S6	X	X	889.4182	800.5264	0.59
**Compound 21**	$C_{41}H_{76}N_6O_{11}$	828.5572	828.5528	-5.31	S6	X	X	917.4507	828.5589	2.02
